# An Analytic Hierarchy Process-based case study on older adult-friendly community therapeutic landscape design

**DOI:** 10.3389/fpubh.2025.1736308

**Published:** 2026-01-13

**Authors:** Yan Han, Bin Li, Juan Zhang

**Affiliations:** 1 Department of Spatial Culture Design, Graduate School of Techno-Design, Kookmin University, Seoul, Republic of Korea; 2 Faculty of Human Ecology, Universiti Putra Malaysia, Serdang, Selangor, Malaysia; 3 Department of Industrial Design, College of Art and Design, Beijing University of Technology, Beijing, China

**Keywords:** therapeutic landscapes, Stress Reduction Theory, Attention Restoration Theory, age-friendly community, Analytic Hierarchy Process

## Abstract

With the accelerating trend of global population aging, the demand for social services among older adults continues to rise. Community therapeutic landscapes play a critical role in promoting the physical and mental well-being of older adults, fostering social interactions, enhancing living environments, and contributing to the sustainable development of communities. Grounded in Stress Reduction Theory (SRT) and Attention Restoration Theory (ART), this study identifies and synthesizes the design characteristics of therapeutic landscapes and subsequently develops an evaluation framework for community therapeutic landscape design cases. Utilizing the Analytic Hierarchy Process (AHP), a hierarchical evaluation model for therapeutic landscapes is developed, and the relative weights of key indicators are calculated. The results indicate that the Longfor Yinian Apartment Therapeutic Garden performs best in activity convenience, spatial adaptability, and social facilitation; the Elizabeth & Nona Evans Therapeutic Garden excels in natural attractiveness, exploration and interest, and environmental tranquility; and the Sun City Kobe Retirement Residence demonstrates notable strengths in environmental tranquility and natural attractiveness. Overall, contemporary community therapeutic landscape design for older adults shows stronger performance in natural attractiveness and environmental tranquility, while social facilitation remains comparatively underdeveloped. Future practice should therefore further reinforce the planning and construction of social spaces to build a more comprehensive and balanced therapeutic landscape system.

## 1 Introduction

Advances in medical care and educational attainment have extended human life expectancy, leading to year-on-year growth in the global older adult population. Population aging has thus become a major challenge confronting countries in the twenty-first century. According to World Population Prospects 2024, by the late 2070s the number of people aged 65 years and older will reach 2.2 billion, surpassing—for the first time—the population under 18 years of age (1). Population aging is associated with a higher prevalence of multimorbidity and cognitive impairment, as well as mental health conditions such as depression and anxiety, thereby increasing demand for long-term care and health services (2–4). However, conventional long-term care institutions and healthcare facilities offer only partial coverage and remain insufficient to meet the needs of the large and growing population of older adults. To address this challenge, community therapeutic landscapes have gradually entered public awareness as a complementary form of healthcare. As the primary spatial unit of daily life for older adults, the community plays an irreplaceable role in enhancing physical and mental health, facilitating social interaction, and improving the residential environment (5, 6). The pronounced health-promoting effects, ease of use, and social functions of landscape spaces within the community have enabled therapeutic landscapes built upon these spaces to gradually emerge as a new approach to meeting the health needs of older adults and enhancing social equity (7).

However, the development of theories and practices related to community therapeutic landscapes has been uneven (8). Therapeutic landscapes, derived from theories in environmental psychology, health geography, medicine, and environmental science, have received widespread attention and recognition from various sectors of society since the concept was first proposed. Grounded in environmental psychology, Stress Reduction Theory (SRT) and Attention Restoration Theory (ART) constitute the coupled theoretical foundation of therapeutic landscapes, explaining the mechanisms through which natural and landscape environments influence individuals’ physical and mental health from the perspectives of reducing physiological and psychological stress and restoring attention, respectively (9, 10). In the 1990s, building on the field of health geography, Gesler proposed the concept of “therapeutic landscapes,” after which theoretical and empirical studies on the relationship between landscape and health gradually became a focal point of interdisciplinary research (11). In terms of theoretical research, scholars from various fields have extended and elaborated the concept of therapeutic landscapes from the perspectives of holistic medicine, geography, and environmental science, emphasizing the relationships between people and environments, perception and environments, and experience and environments (12, 13). In terms of empirical research, a series of epidemiological and public health studies has demonstrated that the quantity and quality of accessible green spaces around residential areas are closely associated with residents’ mental health and self-rated health (14). Exposure to natural environments and participation in horticultural activities can help reduce agitation, improve sleep, and decrease medication use among older adults (15). Renovated therapeutic gardens have been shown to increase the frequency of use, social behaviors, and the ability to spend time outdoors independently among older adults with dementia, while significantly alleviating anxiety, depressive symptoms, and agitation (16). Despite the substantial body of research on the health benefits of landscapes, most studies still remain at the level of providing theoretical arguments and empirical evidence that nature is beneficial to health, while research that directly addresses the relationships among community landscape spaces, older adults, and design practice remains relatively limited. Moreover, a substantial gap still exists between evidence-based research and concrete landscape design recommendations, and studies that further translate such evidence into site design, comparative case analyses, and the development of design guidelines remain even more limited (17, 18).

To address these gaps, this study is primarily guided by the following objectives:

1. To refine the environmental characteristics of therapeutic landscapes within the theoretical framework of Stress Reduction Theory (SRT) and Attention Restoration Theory (ART), and to construct a dimensional framework for evaluating therapeutic landscapes.
2. To introduce the Analytic Hierarchy Process (AHP) in order to determine the relative weights of each evaluation dimension and indicator, thereby establishing a systematic evaluation index system.
3. To select typical design cases for evaluation and comparative analysis, with the aim of revealing the commonalities and differences in community therapeutic landscape design for older adults across different contexts, summarizing the strengths and weaknesses of existing designs, and proposing targeted directions for optimization.

This study aims to clarify how therapeutic landscape characteristics centered on Stress Reduction Theory (SRT) and Attention Restoration Theory (ART) can be concretely translated into design practice, thereby providing theoretical support and practical guidance for the scientific evaluation, design strategies, and future development of community therapeutic landscapes for older adults.

## 2 Theoretical foundations

### 2.1 Therapeutic landscape theory

In 1984, environmental psychologist Roger Ulrich, in his seminal paper “View through a Window May Influence Recovery from Surgery,” was the first to empirically demonstrate the positive effects of landscapes on human health (9). Building on this work, in 1991 he further proposed that exposure to natural environments has restorative effects on both physiological and psychological states (19). In 1992, Wilbert M. Gesler introduced the concept of “Therapeutic landscapes,” defining them as places that promote healing and enhance well-being through the combined effects of physical, social, and symbolic factors within specific geographic settings. Gesler emphasized that healing does not occur exclusively in hospitals or medical institutions, but can also take place in natural or built environments imbued with symbolic and sociocultural meaning. He advocated for understanding health through the lens of “sense of place,” arguing that health is not solely determined by biomedical factors but is shaped by culturally embedded forms of care. Through interactions with particular environments and places, individuals are encouraged to engage in health-promoting behaviors (10). Expanding on this idea, in 1993, Gesler refined the definition of therapeutic landscapes, describing them as “landscape environments capable of fostering healing across physical, psychological, and spiritual dimensions (20).” Concurrently, Allison Williams defined therapeutic landscapes as places explicitly associated with treatment and rehabilitation, aimed at promoting healing across physical, psychological, and spiritual dimensions through the physical and psychological environment (21). Moreover, Gesler emphasized that landscapes with a strong sense of place play a crucial role in sustaining and enhancing individual and collective health and well-being. Overall, therapeutic landscapes can be understood as spaces grounded in natural environments that integrate social structures, place-based culture, and healthcare elements, and that exert restorative effects on human health.

### 2.2 Stress reduction theory

In 1983, within a psychoevolutionary framework, Roger Ulrich proposed the Stress Reduction Theory (SRT), also known as the “Psychoevolutionary Theory” (22). This theory posits that, over the course of biological evolution, humans exhibit a psychological tendency to rely on and prefer certain natural features in response to stress, recognizing that natural environments facilitate psychological stress relief (23). It emphasizes that environmental design should integrate natural elements to reduce mental stress (24) (Table 1). Ulrich’s Stress Reduction Theory (SRT) has been widely applied in contemporary environmental design—particularly in the design of hospitals, landscapes, educational settings, and residential care and rehabilitation facilities—thereby advancing a deeper understanding of the relationship between natural environments and human health (25, 26).

**Table 1:** Environmental characteristics under the stress reduction theory.

Environmental characteristics	Characteristic description
Sense of control	Individuals with a sense of control experience lower stress levels, demonstrate greater adaptability to stress, and tend to have better overall health compared to those who lack control.
Social support	Individuals receive psychological, material, or physical support from other individuals or groups.
Physical activity and exercise	Physical activity benefits both mental and physical health.
Nature-based interventions	Positive interventions refer to actions that enhance or improve a patient’s emotional and cognitive state while preventing or reducing distressing thoughts.

### 2.3 Attention restoration theory

Environmental psychologists Rachel Kaplan and Stephen Kaplan introduced “Attention Restoration Theory (ART),” which focuses on alleviating attentional fatigue through environmental exposure, referring to such settings as “restorative environments” (27, 28). The core concepts of this theory include “directed attention,” “involuntary attention,” and “attentional fatigue,” building upon William James 1892 distinction between voluntary attention and involuntary attention (29, 30). Building upon this framework, the Kaplans delineated four essential attributes of restorative environments: being away, extent, fascination, and compatibility (Table 2).

**Table 2:** Characteristics of the environment under the attention restoration theory.

Environmental characteristics	Characteristic overview
Being away	Refers to distancing oneself from sources of fatigue or stress, providing a space for escape and relaxation.
Extent	Refers to a sufficiently expansive space that fosters a sense of distance from the surrounding environment.
Fascination	Refers to an environment or element that is sufficiently captivating to sustain attention over time.
Compatibility	Refers to an environment that aligns with an individual’s goals or inclinations, ensuring coherence and mutual compatibility.

The Stress Reduction Theory (SRT) and Attention Restoration Theory (ART) exhibit both differences and interconnections. SRT highlights the potential health benefits of natural environments, arguing that individuals responses to nature are rooted in evo-lutionary instincts, with a primary focus on physiological and psychological reactions to stress-inducing situations. In contrast, ART posits that exposure to nature involves not only unconscious responses but also conscious cognitive engagement, addressing the depletion of directed attention caused by the cognitive demands of daily life and work. Although these two theories examine the effects of the environment on human health from different perspectives, they are not mutually exclusive and, in essence, both are theoretical generalizations of restorative environments based on humans’ innate affinity for nature (31). They merge with each other to form a complementary theoretical framework for understanding the relationship among nature, humans, and health, thereby providing a theoretical basis for the further development of therapeutic landscapes.

### 2.4 Characteristic dimensions of therapeutic landscapes

Based on the similarities and differences between the Stress Reduction Theory (SRT) and Attention Restoration Theory (ART) in design applications, the key characteristics of therapeutic landscape design can be identified. Analyzing these characteristics reveals that the environmental factors emphasized in SRT (namely, sense of control, social support, physical activity and exercise),and natural environmental interventions share conceptual parallels with the attributes defined in ART, such as being away, extent, fascination, and compatibility. This suggests a degree of convergence and mutual reinforcement between the environmental principles underlying both theories (22, 28, 32). This study reviews and compares the environmental characteristics of therapeutic landscapes derived from Stress Reduction Theory (SRT) and Attention Restoration Theory (ART) and, following the principles of objectivity, representativeness, systematicity, and exclusivity, classifies and integrates the relevant characteristics to further distill the core environmental characteristics of therapeutic landscapes.

In natural environments, there are many sources of soft fascination, such as the movement of leaves, the shimmer of water surfaces, and changes in clouds, all of which can help relax the mind to some extent (33). At the theoretical level, this can be regarded as the result of the combined effects of the two environmental characteristics of Nature-Based Interventions and Fascination. Compared with urban environments, natural environments more readily evoke a sense of being away and psychological detachment. By enabling people to temporarily escape from sources of stress, they allow individuals to feel relaxed and to alleviate fatigue more quickly (34, 35). At the theoretical level, this type of environmental characteristic can be well explained by the two environmental characteristics of Nature-Based Interventions and Being Away. At the same time, experimental studies have shown that, after attention has been depleted, short-term exposure to natural scenes with high levels of fascination and extent can significantly restore attention (36). At the theoretical level, this can be regarded as a concentrated manifestation of the environmental characteristics of Nature-Based Interventions, Fascination, and Extent. Restorative experiences depend not only on the natural attributes of the environment itself but are also strongly shaped by the social context. Studies have found that older adults tend to prefer therapeutic environments that support mild social interaction (37). This implies that such places need to embody the environmental characteristic of Social Support while also exhibiting good Compatibility with users’ preferences. Spaces with high spatial compatibility offer considerable convenience for people. Research has shown that, in order to achieve such high spatial compatibility, it is necessary to provide, within a certain spatial extent, opportunities for people to choose routes, stopping points, and modes of activity, thereby enhancing their Sense of Control and better meeting their needs and preferences (38). At the theoretical level, this can be interpreted as an expression of the environmental characteristics of Sense of Control, Compatibility, and Extent. Moreover, multiple empirical studies and integrated research findings have indicated that short-term physical activity in natural environments can significantly improve mood, lower blood pressure, and reduce depressive and anxious symptoms, and that it is suitable for different age groups (39). This helps to explain the effectiveness of the environmental characteristic of Physical Activity and Exercise in promoting health.

Based on the above discussion, the following core environmental characteristics of therapeutic landscapes can be derived: Natural Attraction (Nature-Based Interventions, Fascination), Environmental Serenity (Nature-Based Interventions, Being Away), Explorability & Fascination (Nature-Based Interventions, Fascination, Extent), Social Supportiveness (Social Support, Compatibility), Spatial Compatibility (Sense of Control, Compatibility, Extent), and Activity Accessibility (Physical Activity and Exercise) (Table 3) (40).

**Table 3:** Summary of therapeutic landscape characteristic dimensions.

Characteristic dimensions	SRT	ART	Design considerations
Natural attraction	Natural landscapes reduce stress hormones and blood pressure, facilitating physiological and psychological recovery.	The natural environment restores mental fatigue through involuntary attention, providing a sense of relaxation.	Provide visually appealing natural landscapes (e.g., dynamic water features, high greenery coverage, and colorful vegetation) to facilitate sensory relaxation and attention restoration through natural elements.
Environmental serenity	Emphasizing how natural landscapes help individuals distance themselves from stressors, creating a sense of “escape.”	Provides a space away from daily stressors, aiding attention restoration.	Design quiet zones with noise isolation (e.g., green buffers, small courtyards) to create healing spaces that offer a sense of privacy and security.
Explorability & fascination	Drawing attention through engaging landscape design to evoke interest and alleviate stress.	Offers a sense of spaciousness and employability, fostering fascination and restoring attention.	Design winding pathways and multifunctional zones, incorporating dynamic and seasonal landscape features (e.g., seasonal plants, and waterfalls) to stimulate older adults’ exploratory interest.
Social supportiveness	Emphasizing the importance of social interaction and emotional support to facilitate psychological restoration.	Not mentioned directly, but “compatibility” may reflect an environment that matches the needs of the user, indirectly supporting human interaction.	Create public spaces suitable for older adults’ gatherings (e.g., benches, activity areas) to foster social interactions, enhance a sense of belonging, and provide psychological support.
Spatial compatibility	Spatial design should accommodate the needs of older adults (barrier-free and highly accessible), enabling better control and adaptation to the environment.	Design should be compatible with users’ behavioral and psychological needs, minimizing conflicts between the environment and its users.	Provide wheelchair-accessible design, clear pathway planning, and an easily recognizable wayfinding system to ensure ease of use for the older adults.
Activity accessibility	Alleviate stress through physical exercise and activities.	Not mentioned.	Provide low-intensity fitness facilities, walking trails, slip-resistant surfaces, and age-friendly activity spaces to encourage gentle exercise and promote health.

## 3 Materials and methods

### 3.1 Study areas

This study adopts two levels, the “regional level” and the “project level,” to progressively screen and determine the case studies. At the regional level, countries and regions are selected based on a comprehensive consideration of the development of therapeutic landscapes research and the characteristics of population and social background. The United States, as a pioneering country in the field of therapeutic landscapes, demonstrates significant advantages in both publication volume and research centrality (8). Its strong theoretical foundation has substantially contributed to the advancement of design practices. In terms of aging populations, China, as the most populous country in the world, also has the largest number of older adults. By the end of 2024, the population aged 65 and above in China reached 220 million (41). Meanwhile, in Japan, this age group accounts for approximately one-third of the total population, making it the most aged society globally (42). This study selects the United States, China, and Japan as representative case countries to ensure that the findings possess a certain degree of generalizability and representativeness in the context of aging societies. At the project level, design cases must meet the following criteria: (1) older adults are the primary target group; (2) the project explicitly emphasizes therapeutic and healing goals in relation to health; (3) it provides services for urban residents and surrounding communities and is relatively typical or exemplary within its regional context; (4) it has been completed and put into use and has relatively complete, publicly accessible documentation to enable systematic evaluation and expert judgment.

Based on the above criteria, this study selects the Elizabeth & Nona Evans Restorative Garden as the representative case in the United States (43). This project is specifically designed for older adults and people with functional limitations, integrating horticultural therapy, sensory experiences, and barrier-free walking spaces, and it provides services for the urban public and surrounding community residents. At the same time, it is a typical and representative example of therapeutic landscape practice in North America, and relatively complete graphic and textual documentation is available through the American Society of Landscape Architects (ASLA) website.^1^ In China, this study selects the Longfor Yinian Apartment Therapeutic Garden in Chongqing as the representative case (44). The project is built on the rooftop platform of an urban senior apartment complex and primarily provides outdoor spaces for resident older adults that integrate daily walking, rehabilitation exercise, and social activities. It is one of the typical examples of integrated practice combining community-based older adult care and therapeutic landscapes in the context of high-density Chinese cities. The project is also well documented: its plan layout, design description, photographs, and video materials can be accessed on the gooood platform, which focuses on outstanding architectural and landscape design projects worldwide.^2^ In addition, this study takes the therapeutic garden of Sun City Kobe Retirement Residence as the typical design case for Japan (45, 46). This therapeutic garden is built in association with a large residential and nursing complex for older adults, and organizes walking, resting, and social spaces through a central courtyard and surrounding gardens. It represents a typical model in Japan’s aging society that integrates living, care, and outdoor therapeutic environments. Relatively complete plans and photographic documentation can be obtained from two professional architectural and landscape case-study platforms, mooool^3^ and ARCHINA^4^ (Table 4). It is also worth noting that the approximately 46,451 m^2^ reported for the Sun City Kobe Retirement Residence therapeutic garden in Japan refers to the land area of the entire older adult care complex, whereas the areas of the other two cases refer only to their core therapeutic garden spaces. This study does not use total site area as an evaluation indicator. Instead, it primarily examines how the design cases employ therapeutic landscape design strategies and how they express the restorative characteristics of the environment.

**Table 4:** Detailed overview of therapeutic landscape cases.

**Case**	**Location**	**Area**	**Design concept**	**Source for original visuals (website)**
Elizabeth & Nona Evans Therapeutic Garden	Ohio, USA	1115 m^2^	Integrating education, social responsibility, culture, and environmental stewardship, the design fosters a positive relationship between users and plants. Through sensory experiences and educational elements, it offers a thoughtful and enjoyable service.	American Society of Landscape Architects (ASLA) awards page (full URL listed in References) (43)
Longfor Yinian Apartment Therapeutic Garden	Chongqing, China	890 m^2^	By encouraging older adults participation in gardening, rehabilitation exercises, and social activities, the design enhances well-being and a sense of belonging, ultimately improving quality of life. It advocates the concept of a "benevolent landscape," addressing both the physical and psychological needs of the older adults to create a warm and supportive care environment.	Gooood (Design media platform) project page (full URL listed in References) (44)
Sun City Kobe Retirement Residence	Kobe, Japan	46451 m^2^ (Project Area)	The design centers on "harmonious coexistence between humans and nature," aiming to enhance the quality of life for older adults through natural landscapes, emphasizing therapeutic functions, barrier-free accessibility, and social spaces.	Mooool project page; Archina project page (full URLs listed in References) (45, 46)

This table provides an author-prepared textual and structured summary of each case. For complete visual materials and source details (including full URLs and access dates), please refer to the corresponding entries in the reference list.

These three cases, located in North America and East Asia respectively, encompass diverse geographical and cultural contexts, thus constituting a representative sample of therapeutic landscape design from a cross-regional perspective. This facilitates the extraction of common principles and distinctive characteristics. Through comparative analysis of these international cases, this study aims to provide diverse experiential references and theoretical support for the further development of therapeutic landscape theory and practice.

### 3.2 Research methods and procedures

This study intends to evaluate design cases of community therapeutic landscapes for older adults and, based on the evaluation results, to derive the current state of practical development in this field and clarify its strengths and weaknesses. Because this study essentially concerns a decision-making problem built upon multiple design dimensions, it is difficult to assess the overall problem using a single indicator. Moreover, since the study as a whole involves a high level of professional and theoretical complexity, it is not appropriate to conduct the research through large-sample questionnaires or user-based quantitative data. Instead, it should primarily rely on the professional judgments of a small number of experts. For this reason, conventional statistical and modeling methods are not suitable. AHP, a widely used systematic method for multi-criteria decision-making, was introduced by American operations researcher Thomas L. Saaty in the 1970s (47). Particularly well-suited for complex and multidimensional problems, the Analytic Hierarchy Process (AHP) excels in decomposing intricate issues into a hierarchical structure, quantifying the relative importance of decision factors, and providing a clear prioritization scheme. By structuring subjective judgments through mathematical methods, AHP enhances consistency, transparency, and comparability in comprehensive evaluation processes (48, 49). Therefore, the Analytic Hierarchy Process (AHP) is considered capable of structuring design dimensions into a hierarchical system and, through comparative scoring by experts, transforming qualitative judgments into quantitative analysis. In addition, AHP has clear advantages in obtaining weights from small expert samples, making it suitable for constructing the evaluation framework and weighting system for community therapeutic landscapes for older adults (50).

It should be noted that classical AHP theoretically assumes that the evaluation criteria at the same level are independent of one another (51). To approximate this assumption as closely as possible, this study, when constructing the index system, first conducted a systematic review of therapeutic landscape–related literature based on Stress Reduction Theory (SRT) and Attention Restoration Theory (ART), and defined each first-level dimension as a conceptually relatively independent design characteristic, so that each criterion corresponds, as far as possible, to a single and clearly defined design meaning. At the same time, in real-world community therapeutic landscapes, it is difficult for different design elements to be completely independent of one another. For example, places with higher spatial compatibility are often also more favorable for activity accessibility. Therefore, the weights obtained in this study are better understood as a ranking of relative importance based on the current theoretical framework and expert judgments, rather than as precise estimates under a strict assumption of statistical independence. Furthermore, AHP has been extensively utilized in existing research to evaluate multi-criteria age-friendly community spaces (52–54).

**Figure 1:**
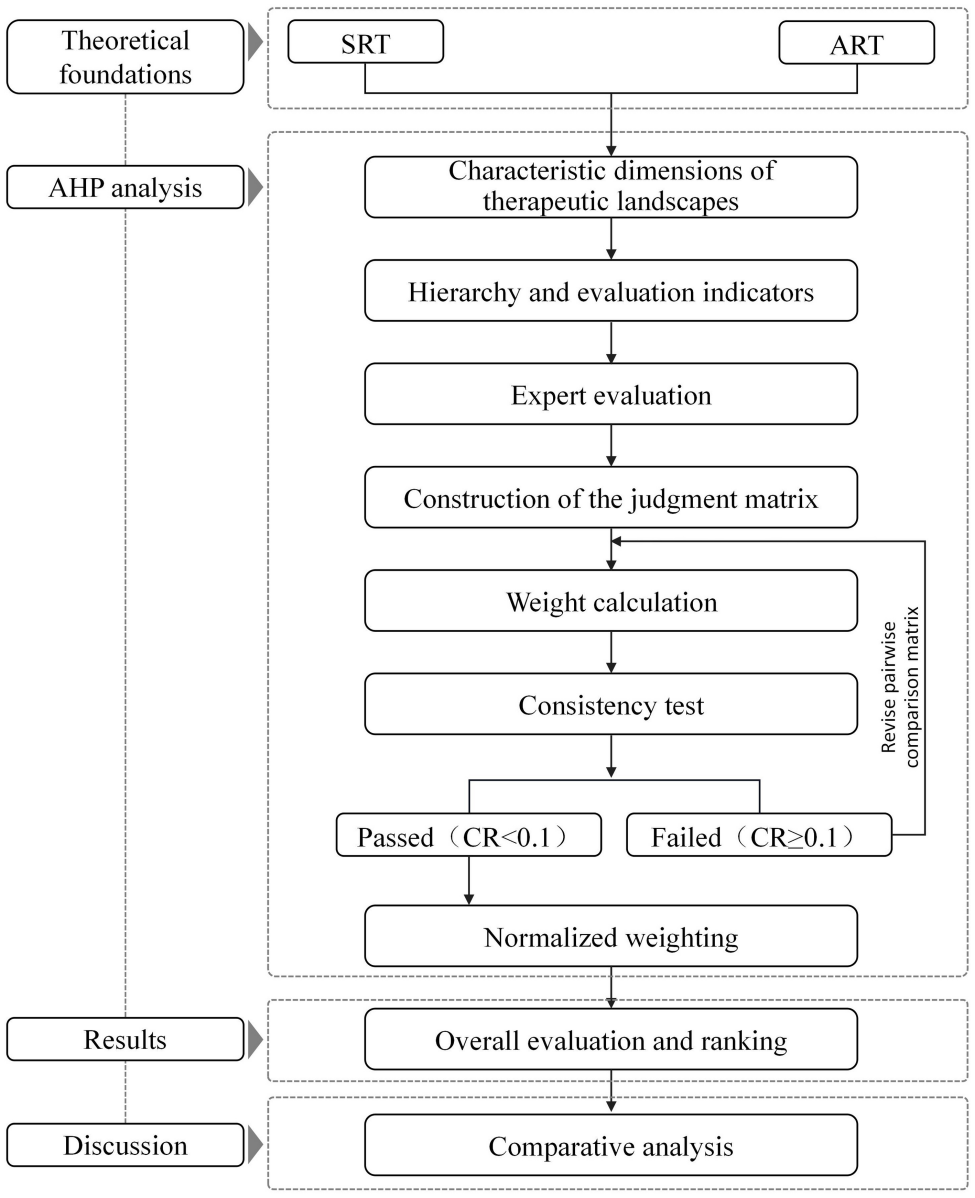
Case study framework diagram.

To identify the relative strengths and weaknesses of each design case, the study was conducted through the following main steps (Figure 1).

#### 3.2.1 Establishment of the hierarchical structure

First, based on the characteristic dimensions of therapeutic landscapes, a hierarchical structure was established, consisting of a goal level, a criterion level, and a sub-criterion level. The establishment of this hierarchy followed the principles of systematic organization, clear hierarchy, and mutual independence, ensuring logical clarity and avoiding redundancy or overlap among elements, thereby minimizing subjective interpretation bias from the outset (55).

#### 3.2.2 Construction of the judgment matrix

Multiple experts from relevant research fields are invited to evaluate the importance of the criteria and sub-criteria, and to construct the corresponding judgment matrix $A=[a_{\mathrm{ij}}]$ , Each element in the matrix represents the relative importance of the i-th criterion compared to the j-th criterion. According to the fundamental principles of AHP, the matrix satisfies the condition $a_{\mathrm{ii}}$ =1, and the reciprocal property $a_{\mathrm{ii}}$ =1/ $a_{\mathrm{ji}}$ . Meanwhile, the evaluation adopts the Saaty 1–9 scale, where 1 indicates equal importance, 3 indicates slight importance, 5 indicates moderate importance, 7 indicates strong importance, and 9 indicates extreme importance; 2, 4, 6, and 8 represent intermediate values (56).

#### 3.2.3 Weight calculation and consistency check

To calculate the relative weights of each evaluation criterion, this study employs the eigenvector method in AHP. By solving the characteristic equation $A\cdot \omega =\lambda_{\max}\cdot \omega$ the eigenvector ω is obtained. After normalization, the eigenvector serves as the weight vector of the criteria, where $\lambda_{\max}$ denotes the maximum eigenvalue of the judgment matrix. Subsequently, to ensure the logical consistency, rationality, and reliability of the expert evaluations, a consistency check of the judgment matrix is required (57–59). Specifically, the Consistency Index (CI) and Consistency Ratio (CR) are calculated, with the formulas given as follows:


$$\mathrm{CI}=\frac{\lambda_{\max}-n}{n-1},\mathrm{CI}=\frac{\mathrm{CI}}{\mathrm{RI}}$$

where CI denotes the Consistency Index, λmax is the maximum eigenvalue of the judgment matrix, and n is the order of the matrix. CR refers to the Consistency Ratio, and RI is the Random Index, obtained from the standard table provided by Saaty (Table 5) (58). When CR < 0.1, the consistency of the judgment matrix is considered acceptable. If CR ≥ 0.1, the judgment matrix must be adjusted, the weights recalculated, and the consistency check repeated until the requirement is satisfied.

**Table 5:** Reference values of RI (Random Index).

N	1	2	3	4	5	6	7	8	9	10	11	12	13	14	15
RI	0.00	0.00	0.52	0.89	1.12	1.26	1.36	1.41	1.46	1.49	1.52	1.54	1.56	1.58	1.59

#### 3.2.4 Overall evaluation and ranking of design cases

After completing the construction of the hierarchical structure and the calculation of indicator weights, this study subsequently undertakes a comprehensive evaluation of the three design cases of community therapeutic landscapes for older adults. Owing to geographical constraints, the experts were required to base their judgments on graphical and textual materials available online. To minimize interference arising from fragmented online impressions and variations in information quality across different websites, this study standardized the evaluation materials for each case (including plans, representative spatial photographs, and design descriptions) prior to scoring, explicitly excluding built-in star ratings and visitor comments on the websites, in order to enhance the degree of standardization and consistency of the evidentiary basis for evaluation.

Subsequently, the same panel of experts who participated in determining the AHP weights used the Saaty 1–9 scale to score the performance of each design case with respect to the indicators at the sub-criteria level. Higher scores indicate that a given case performs better on the corresponding sub-criterion. For each sub-criterion i and case *k*, the experts’ scores were averaged arithmetically to obtain the group score $r_{\mathrm{ik}}$ . When constructing the calculation table, the scores of the three cases under each sub-criterion were further normalized so that their sum was equal to 1. On this basis, the previously obtained global weights $\omega_{i}$ for the sub-criteria were multiplied by the corresponding group scores $r_{\mathrm{ik}}$ and then summed (60) to obtain the composite score of case *k*:


$$S_{k}=\sum_{i}\omega_{i}\cdot r_{\mathrm{ik}}$$

On this basis, the three design cases were ranked to clarify their relative performance within the system of therapeutic landscape characteristics and to provide a basis for the subsequent optimization of community therapeutic landscape design for older adults.

### 3.3 Evaluation indicators and data collection

**Figure 2:**
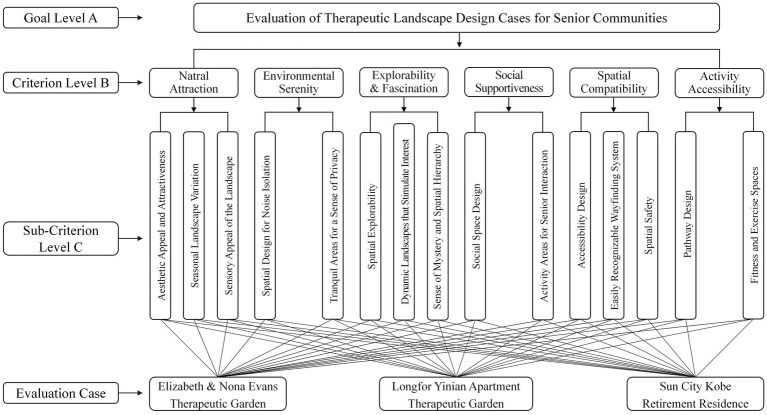
Hierarchical structure.

Based on the characteristic dimensions of therapeutic landscapes derived from Stress Reduction Theory (SRT) and Attention Restoration Theory (ART), this study constructed an evaluation indicator system for community therapeutic landscapes for older adults (Table 6). Using the Analytic Hierarchy Process (AHP), “Evaluation of community therapeutic landscape design cases for older adults” was set as the goal level, six therapeutic landscape dimensions served as the criterion level, and the design elements within each dimension were organized into the sub-criterion level, thereby forming a systematic and logically coherent hierarchical model. This model not only presents the primary dimensions and specific indicators for evaluating therapeutic landscape design cases, but also delineates a complete hierarchical structure (Figure 2).

**Table 6:** Hierarchical evaluation model for therapeutic landscape design cases in senior communities.

Goal level A	Criterion level B	Sub-criterion level C	Factor description
A1 Evaluation of Therapeutic Landscape Design Cases for Senior Communities	B1 Natural Attractiveness	C1 Aesthetic Appeal and Attractiveness	Green coverage ratio, dynamic water features, etc.
		C2 Seasonal Landscape Variation	Seasonal plants
		C3 Sensory Appeal of the Landscape	Color richness, visual interest, and soundscape
	B2 Environmental Tranquility	C4 Spatial Design for Noise Isolation	Green buffer
		C5 Tranquil Areas for a Sense of Privacy	Such as small courtyards and resting corners
	B3 Exploration and Interest	C6 Spatial Explorability	Winding pathways and diverse functional zones
		C7 Dynamic Landscapes that Stimulate Interest	Such as flowing water and sculptures
		C8 Sense of Mystery and Spatial Hierarchy	Secluded corners and visual transition design
	B4 Social Facilitation	C9 Social Space Design	Benches, plazas, and public activity areas
		C10 Activity Areas for Senior Interaction	Such as community gardens and handicraft areas
	B5 Spatial Adaptability	C11 Accessibility Design	Ramps, wheelchair accessibility, handrails, etc.
		C12 Easily Recognizable Wayfinding System	Signage, directional ground markings, etc.
		C13 Spatial Safety	Well-designed lighting and roadway systems
	B6 Activity Accessibility	C14 Pathway Design	Non-slip surfaces, appropriate length and width
		C15 Fitness and Exercise Spaces	High-, medium-, and low-intensity exercise equipment, various types of activity spaces (e.g., sports courts, square dance areas, etc.)

This study is based on international case studies. Due to spatial constraints, site-specific information was obtained from publicly accessible websites (ASLA, gooood, mooool), while expert evaluation data were collected as primary data. To ensure the professionalism and scientific rigor of the case evaluations, 11 experts (including professors and doctoral researchers) were invited to participate in the judgment matrix scoring. The expert panel was composed of individuals from diverse disciplinary backgrounds: four experts in landscape and urban design, two in geriatrics and rehabilitation, three in community governance and spatial studies, and two in public health and health promotion (Supplementary Table 1). This structure was intended to ensure professional quality, interdisciplinary representation, and practical relevance in the evaluation results. Furthermore, group-based expert evaluations help reduce subjective bias caused by individual experience or personal preferences.

Based on expert evaluations of criteria and sub-criteria, the comprehensive weight calculations reveal the strengths and weaknesses of each case. Specific directions for design optimization are proposed according to the evaluation results. Through this approach, the study achieves a transition from qualitative analysis to quantitative evaluation, thereby providing a scientific foundation for the assessment of therapeutic landscape design.

## 4 Results

To ensure the logical consistency, rationality, and credibility of the expert scoring results, the judgment matrices for the goal level A, the criteria level B, and the sub-criteria level C were subjected to a consistency test, and the results are shown in Table 7. Except for matrices with dimension n = 2 and RI = 0, the CR values of all other judgment matrices are all less than 0.1, meeting the consistency requirement of AHP. In addition, for matrices with n = 2, since CI = 0, they can be regarded as perfectly consistent. On this basis, the factor weights of the evaluation indicators at each level of the AHP hierarchy for community therapeutic landscape design for older adults were calculated, and the final overall weights of the indicator factors at the Goal Level, Criterion Level, and Sub-Criterion Level were obtained, as reported in Table 8.

**Table 7:** Summary of consistency diagnostics.

Judgment matrix	Dimension *n*	λ^max^	CI	RI	CR	Consistency test result
Goal Level A	6	6.02	0.004	1.26	0.003	Passed
Criterion Level B1	3	3.001	0	0.52	0.001	Passed
Criterion Level B2	2	2	0	0	N/A	Passed
Criterion Level B3	3	3.001	0.001	0.52	0.001	Passed
Criterion Level B4	2	2	0	0	N/A	Passed
Criterion Level B5	3	3	0	0.52	0	Passed
Criterion Level B6	2	2	0	0	N/A	Passed
Sub-Criterion Level C1	3	3	0	0.52	0	Passed
Sub-Criterion Level C2	3	3.001	0.001	0.52	0.001	Passed
Sub-Criterion Level C3	3	3	0	0.52	0	Passed
Sub-Criterion Level C4	3	3.001	0	0.52	0.001	Passed
Sub-Criterion Level C5	3	3	0	0.52	0	Passed
Sub-Criterion Level C6	3	3.005	0.003	0.52	0.005	Passed
Sub-Criterion Level C7	3	3.002	0.001	0.52	0.002	Passed
Sub-Criterion Level C8	3	3.007	0.003	0.52	0.007	Passed
Sub-Criterion Level C9	3	3.002	0.001	0.52	0.001	Passed
Sub-Criterion Level C10	3	3.003	0.002	0.52	0.003	Passed
Sub-Criterion Level C11	3	3	0	0.52	0	Passed
Sub-Criterion Level C12	3	3.001	0.001	0.52	0.001	Passed
Sub-Criterion Level C13	3	3	0	0.52	0	Passed
Sub-Criterion Level C14	3	3	0	0.52	0	Passed
Sub-Criterion Level C15	3	3.052	0.026	0.52	0.05	Passed

**Table 8:** Overall allocation table of evaluation index factor weights.

Goal level A	Criterion level			Sub-criteria level			Total weight value
	Criterion level B	Feature vector	Weight	Sub-criterion level C	Feature vector	Weight	
A1 Evaluation of Therapeutic Landscape Design Cases in Senior Communities	B1 Natural Attractiveness	0.394	0.052	C1 Aesthetic Appeal and Attractiveness	0.827	0.2756	0.0144
				C2 Seasonal Landscape Variability	0.655	0.2185	0.0114
				C3 Sensory Appeal of the Landscape	1.518	0.5059	0.0265
	B2 Environmental Tranquility	0.626	0.083	C4 Noise-Reducing Spatial Design	0.651	0.2975	0.0247
				C5 Quiet areas that provide a sense of privacy	1.537	0.7025	0.0584
	B3 Exploration and Interest	0.549	0.073	C6 Explorability of space	1.770	0.5402	0.0394
				C7 Dynamic Landscapes to Stimulate Interest	0.704	0.2150	0.0157
				C8 Mystery and Layers	0.802	0.2449	0.0178
	B4 Social Facilitation	1.603	0.213	C9 Social Space Design	0.691	0.3234	0.0688
				C10 Activity areas suitable for older people’s interaction	1.446	0.6766	0.144
	B5 Spatial Adaptability	1.797	0.239	C11 Accessible design	1	0.3333	0.0795
				C12 Easily Recognizable Wayfinding System	1	0.3333	0.0795
				C13 Space security	1	0.3333	0.0795
	B6 Activity Convenience	2.562	0.34	C14 Trail Design	1.648	0.8242	0.2804
				C15 Fitness and exercise space	0.352	0.1756	0.0598

According to Table 8, at the criterion level (B), the weights of the six dimensions from highest to lowest are as follows: B6 Activity Accessibility (0.34) > B5 Spatial Compatibility (0.239) > B4 Social Supportiveness (0.213) > B2 Environmental Serenity (0.083) > B3 Explorability & Fascination (0.073) > B1 Natural Attraction (0.052). This indicates that safe, continuous, and accessible walking and activity conditions, together with spatial design that matches the physical functional capacities of older adults, are regarded by experts as the two most critical dimensions, whose importance clearly exceeds that of purely landscape aesthetics and visual attraction.

At the sub-criterion level (C), the indicators with relatively higher weights are, respectively, C14 Pathway design (0.2804), C10 Activity areas suitable for interaction among older adults (0.144), C11 Barrier-free design (0.0795), C12 Easily recognizable wayfinding system (0.0795), and C13 Spatial safety (0.0795). These high-weight indicators are mainly concentrated in the three dimensions of Activity Accessibility, Spatial Compatibility, and Social Supportiveness at the criterion level. This indicates that a coherent and appropriately scaled walking system, safe and reliable barrier-free facilities, and public activity spaces that encourage interaction are core elements in constructing community therapeutic landscapes for older adults. This suggests that although landscape aesthetics play a certain facilitating role in restorative environments, functional and interactive design is regarded as a higher priority for promoting the health of older adults.

Through pairwise comparisons conducted by 11 experts across the three design cases, judgment matrices were constructed, and criterion weights were obtained by calculating eigenvectors. All evaluations passed the consistency check (Table 7), indicating that the scoring results were both reasonable and valid (Table 9). By multiplying the weights of the sub-criteria (C1-C15) from Table 9 by the expert scores for each design case, weighted scores were calculated. The final score for each case was determined by summing all weighted scores, thereby reflecting the overall performance of the three cases in the therapeutic landscape evaluation.

**Table 9:** Weight table for therapeutic landscape design cases in senior communities.

Criterion		Weight	Elizabeth & Nona Evans Therapeutic Garden		Longfor Yinian Apartment Therapeutic Garden		Sun City Kobe Retirement Residence	
			Score	Weighted score	Score	Weighted score	Score	Weighted score
B1	C1	0.014	0.451	0.006	0.244	0.004	0.305	0.004
	C2	0.011	0.573	0.007	0.171	0.002	0.256	0.003
	C3	0.027	0.533	0.014	0.242	0.006	0.225	0.006
B2	C4	0.025	0.468	0.012	0.181	0.004	0.351	0.009
	C5	0.058	0.507	0.030	0.157	0.009	0.336	0.020
B3	C6	0.039	0.567	0.022	0.179	0.007	0.254	0.010
	C7	0.016	0.516	0.008	0.206	0.003	0.278	0.004
	C8	0.018	0.574	0.010	0.168	0.003	0.257	0.005
B4	C9	0.069	0.444	0.031	0.313	0.022	0.243	0.017
	C10	0.144	0.376	0.054	0.379	0.055	0.245	0.035
B5	C11	0.080	0.441	0.035	0.409	0.032	0.150	0.012
	C12	0.080	0.215	0.017	0.606	0.048	0.178	0.014
	C13	0.080	0.267	0.021	0.565	0.045	0.168	0.013
B6	C14	0.280	0.257	0.072	0.568	0.159	0.176	0.049
	C15	0.070	0.219	0.015	0.664	0.046	0.116	0.008
Aggregate Rank		-	-	0.354	-	0.446	-	0.209

**Figure 3:**
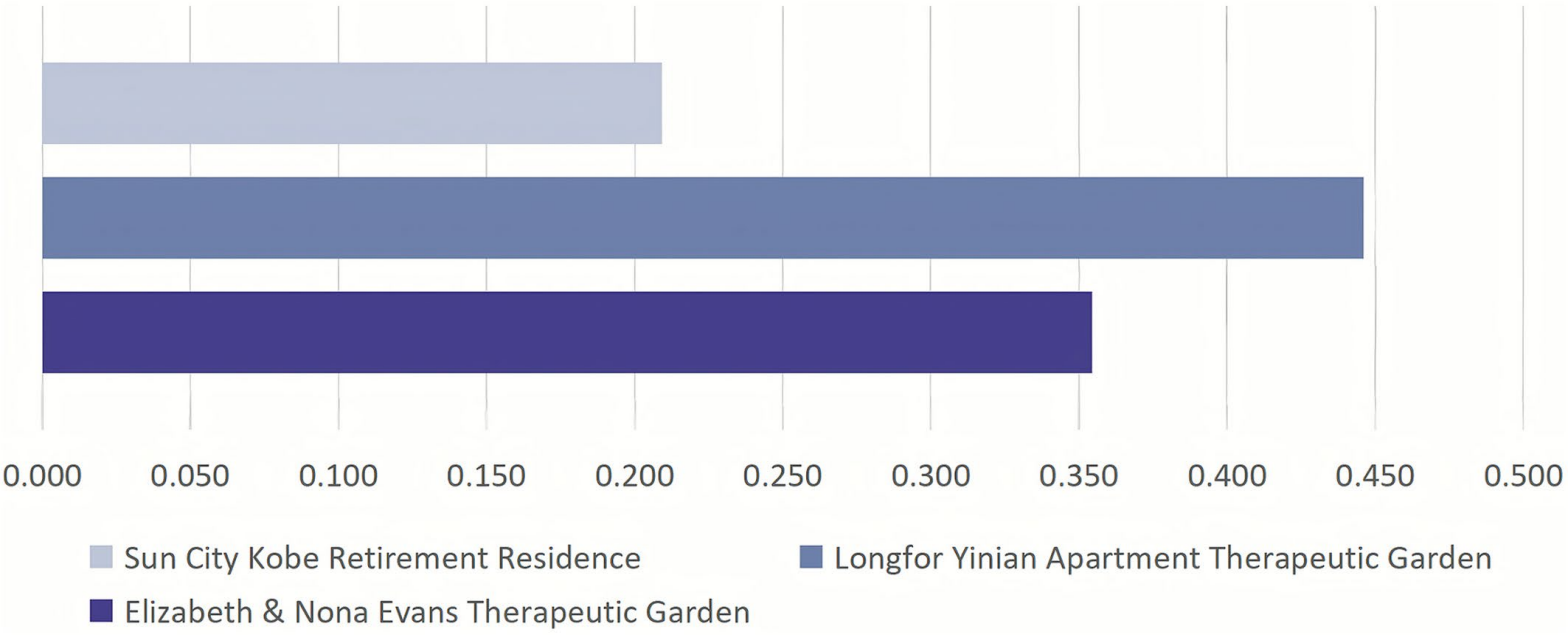
Comprehensive evaluation.

**Figure 4:**
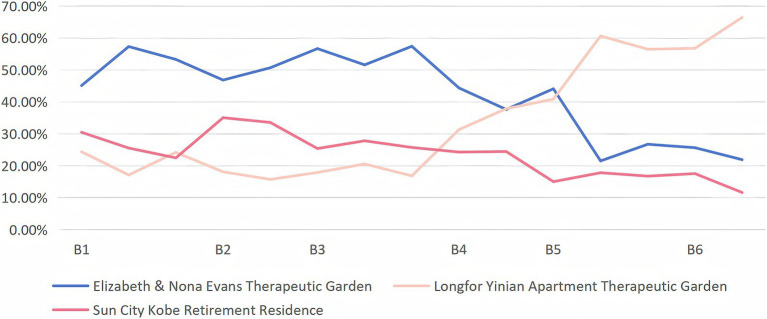
Evaluation at different levels.

The comprehensive evaluation results show the following ranking: Longfor Yinian Apartment Therapeutic Garden > Elizabeth & Nona Evans Therapeutic Garden > Sun City Kobe Retirement Residence (Figure 3). Longfor Yinian Apartment Therapeutic Garden achieved the highest score (0.446), demonstrating outstanding performance in activity convenience, spatial adaptability, and social facilitation. The Elizabeth & Nona Evans Therapeutic Garden ranked second (0.354), with notable strengths in natural attraction and exploration and interest. Sun City Kobe Retirement Residence received the lowest score (0.209); although it performed well in certain sub-criteria such as environmental tranquility and exploration and interest, its overall performance was relatively weaker (Figure 4).

## 5 Discussion

Based on the evaluation data, this study outlines the design strengths of the three case studies, offering a comprehensive understanding of the strategies and methods applied in therapeutic landscape design for senior communities. This provides valuable references for future design efforts. Moreover, the study highlights the weaknesses and limitations inherent in current designs, thereby offering a directional foundation for the future development of therapeutic landscape design.

### 5.1 The design analysis of Longfor Yinian apartment therapeutic garden

Longfor Yinian Apartment Therapeutic Garden (0.446) is the design case with the highest overall evaluation score, with particularly outstanding performance in the three dimensions of Activity Accessibility (B6), Spatial Compatibility (B5), and Social Supportiveness (B4). From a theoretical perspective, it exhibits considerable strengths in providing functions for Physical Activity and Exercise, in calibrating Sense of Control and spatial Extent, and in designing Compatibility and Social Support that align with the physical functions and needs of older adults. This also corroborates the high weights and strong discriminative power of these three dimensions in the AHP results of this study, and constitutes the main reason why this case achieved the highest composite score among the three design cases.

At the Activity Accessibility (B6) dimension, Longfor Yinian Apartment Therapeutic Garden received relatively high evaluations on the sub-indicators C14 Pathway design (0.568) and C15 Fitness and exercise spaces (0.664), indicating that this design case has marked advantages in supporting older adults’ daily walking and functional gait training (Table 10). Previous studies have indicated that excessively long walking distances increase walking-related risks among older adults, and that the lack of resting seats is significantly associated with the duration and frequency of their trips outside the home. Moreover, timely grasping of handrails can reduce impact forces and mitigate injuries in the event of a fall (61–63). The Longfor Yinian Apartment Therapeutic Garden fully embodies key design principles for pathways. The widths and gradients of the paths are carefully calibrated to accommodate wheelchairs and walking aids, ensuring comfortable two-way movement and stopping (≥ 2.40 m), while resting spaces and handrails are provided at key nodes to reduce fatigue and the risk of falls associated with long-distance walking. In terms of the design of fitness and exercise spaces, this case fully takes into account the flatness, shock-absorbing performance, and recognizability of the fitness track, using a colored rubber surface that reduces impact and wear on the knees, ankles, and hip joints while lowering the risk of chronic pain (64). At the same time, the clear color scheme makes the route easier to distinguish, thereby enhancing legibility and a sense of direction along the path (65). In addition, goal setting, self-monitoring, and feedback have been identified as effective intervention strategies for promoting physical activity among older adults (66). Therefore, this design incorporates motivational markings along the exercise track to help older adults set activity goals and self-evaluate their exercise volume. Color plays a positive role in improving the emotional states of older adults and enhancing their cognitive abilities (67). The rainbow pergola in the garden projects rich and constantly changing patterns of light and shadow into the space, significantly enhancing the visual layering and sensory diversity of the environment.

**Table 10:** The design of "activity accessibility" in Longfor Yinian apartment therapeutic garden.

**Activity accessibility B6 sub-criterion**	**Weight**	**Design strategy**	**Health benefit**	**Case description**	**Source for original visuals (website)**
C14	0.568	Accessible walking pathways	Improve circulation freedom and reduce mobility barriers.	Barrier-free looped route with continuous, even paving and adequate width for safe walking.	Gooood project page (full URL listed in References) (44)
C14	0.568	Rubberized running track	Reduce joint impact and wear, lowering the risk of chronic pain.	Shock-absorbing surface supporting low-impact exercise while reducing joint loading.	Gooood project page (full URL listed in References) (44)
C15	0.664	Rainbow trellis	Provides rich sensory stimulation and creates a positive, uplifting atmosphere.	Recognizable landmark canopy providing visual stimulation and supportive wayfinding cues.	Gooood project page (full URL listed in References) (44)
C15	0.664	Motivational prompts	Promote exercise motivation and facilitate appropriate intensity regulation.	Route markings and simple prompts (e.g., distance cues) to encourage participation and pacing.	Gooood project page (full URL listed in References) (44)
C15	0.664	Resting benches with armrests	Improve environmental safety and reduce fall risk due to fatigue or physical discomfort.	Distributed rest points with armrests and suitable seat height to support safe sit-to-stand.	Gooood project page (full URL listed in References) (44)

This table presents an author-prepared textual and structured summary of the case-related design features. For complete visual materials and source details (including the full URL and access date), please refer to the corresponding entry in the reference list (Gooood project page).

In summary, with respect to Activity Accessibility, the Longfor Yinian Apartment Therapeutic Garden provides older adults with a safe and controllable outdoor exercise environment with adjustable physical load through the provision of barrier-free paths, an exercise track, and supporting resting facilities. Overall, the design aligns with the core proposition of Stress Reduction Theory that environmental support for Physical Activity and Exercise helps to relieve tension and reduce stress.

At the dimension of Spatial Compatibility (B5), this design case receives high evaluations on the three sub-indicators C11 Barrier-free design (0.4086), C12 Wayfinding system (0.6064), and C13 Spatial safety (0.5649), with scores generally higher than those of the other two cases (Table 11). This indicates that these accessibility- and safety-related indicators possess strong discriminative power within the present evaluation framework. Existing studies have shown that both an excessive and an insufficient number of steps significantly increase the risk of falls among older adults. In addition, when older adults experience balance impairments and gait changes, ramps are more favorable for movement than stairs, and gentler slopes provide higher levels of safety and comfort for wheelchair use (68, 69). To enhance walking safety for older adults, the Longfor Yinian Apartment Therapeutic Garden minimizes unnecessary steps and replaces level changes with continuous gentle ramps. In addition, to reduce rolling resistance for wheelchair users and improve the safety and comfort of the ramps, the slope is set at a relatively low ratio of 1:12. Clear wayfinding systems, easily recognizable colors and signage, and continuous visual guidance help support spatial orientation and thereby enhance environmental safety and independent walking ability among older adults (70). In this design case, clear route planning, circulation organization, and lighting design are employed to meet the wayfinding needs of older adults. Handrails are an important element for enhancing spatial safety. Appropriate handrail heights facilitate standing up and maintaining stability among older adults, and slightly higher handrails (97–107 cm) help mitigate trunk displacement and improve balance recovery (71). In this design case, to enhance the safety of older adults within the space, a two-level handrail system is adopted. The upper handrail is set at 0.90 m to provide stable support for older adults who are standing and walking, while the lower handrail is set at 0.75 m to facilitate grasping by wheelchair users and to reduce the physical burden during uphill, downhill, or long-distance movement. Although the height of the upper handrail is not the most ideal, it still provides substantial safety protection for older adults. In addition, non-slip paving is widely used on the ground surface, and chamfering is applied to the edges to reduce the potential risks of slipping, tripping, and impact injuries caused by sharp corners.

**Table 11:** The design of "spatial adaptability" in Longfor Yinian apartment therapeutic garden.

**Spatial A daptability B5 sub-criteria**	**Weight**	**Design measures**	**Health benefits**	**Case description**	**Source for original visuals (website)**
C11	0.409	Ramp design	The use of low-step and gently sloped pathways enhances spatial accessibility and perceived safety.	Step-free, gently sloped ramps for safer wheelchair/walker access.	Gooood (Design media platform) project page (full URL listed in References) (44)
C12	0.606	Lighting design	Improve spatial safety.	Pathway and area lighting to improve visibility and perceived safety at night.	Gooood (Design media platform) project page (full URL listed in References) (44)
C13	0.565	Anti-slip paving & chamfered edges	Minimizes the risk of accidental collisions caused by sharp corners.	Anti-slip surfaces and rounded edges to reduce slips and collision risk.	Gooood (Design media platform) project page (full URL listed in References) (44)
C13	0.565	Dual-level handrails	Ensures adequate support for both standing and seated older adults, reducing physical exertion.	Dual-height handrails to support safe movement for varied mobility needs.	Gooood (Design media platform) project page (full URL listed in References) (44)

This table provides an author-prepared textual and structured summary of case-related design features. For complete visual materials and source details (including the full URL and access date), please refer to the corresponding entry in the reference list (Gooood project page).

Longfor Yinian Apartment Therapeutic Garden concretely implements the core requirements of a therapeutic landscape environment through measures such as step-free circulation, gentle ramps, appropriate lighting, and a two-level handrail system. From the perspective of Stress Reduction Theory, which emphasizes that environments should reduce individuals’ perceptions of danger and uncertainty (72), these design strategies enhance older adults’ Sense of Control over the environment, reduce their concerns about falls, and strengthen the overall safety and reliability of the space. From the perspective of Attention Restoration Theory, the above spatial layout is well adapted to the physical functions and everyday behavioral needs of older adults, reflecting a high level of Compatibility between the environment and individual goals. At the same time, the continuous and clearly defined circulation routes and wayfinding system create an overall coherent environment, effectively expressing the environmental characteristic of Extent.

Within the Social Facilitation (B4) dimension, the indicator C10 Activity Areas for Senior Interaction performs particularly well, while C9 Social Space Design, although not superior to the other two design cases, is nevertheless still commendable (Table 12). As a longstanding component of therapeutic landscape design, horticultural therapy has been demonstrated to be an effective way of promoting health through gardening activities. For example, by synthesizing 15 studies, Wang Z. et al. found that horticultural therapy helps improve the physical functioning and quality of life of older adults, reduces body mass index (BMI), and enhances positive emotions and subjective well-being (73). Engaging in gardening activities can effectively improve muscle strength, aerobic endurance, and hand dexterity, while at the same time alleviating symptoms such as depression and anxiety and enhancing cognitive function (74). In the Longfor Yinian Apartment Therapeutic Garden, the design team fully considered the physical conditions and needs of older adults and, within the activity areas for interaction among older adults, installed barrier-free planting beds, vertical planting beds, and multi-tiered planting tables to ensure that all older adults, especially wheelchair users, can conveniently participate in gardening activities and are guided to engage in activities in ways that match their own abilities. Moreover, social activities are not only a means of maintaining social connectedness, but also an important approach to delaying cognitive decline, reducing social isolation, and enhancing subjective well-being (75). To encourage older adults to engage in outdoor viewing and social interaction, the Longfor Yinian Apartment Therapeutic Garden adopts a triangular seating arrangement in its social spaces. This configuration avoids the discomfort associated with direct face-to-face eye contact and enhances psychological comfort during conversation, while also maintaining good visibility of the surrounding landscape so that older adults can appreciate the environment while talking, thereby creating a quiet, pleasant, and visually attractive social setting that helps to alleviate feelings of loneliness.

**Table 12:** The design of "social facilitation" in Longfor Yinian apartment therapeutic garden.

**Social facilitation B4 sub-criteria**	**Weight**	**Design measures**	**Health benefits**	**Case description**	**Source for original visuals (website)**
C9	0.313	Social interaction facilities	Enhances conversational comfort, strengthens environmental social-friendliness, and alleviates loneliness.	Shaded seating and small gathering nodes for conversation.	Gooood (Design media platform) project page (full URL listed in References) (44)
C10	0.379	Horticultural therapy	Promotes social connectedness, reduces loneliness and anxiety, and contributes to psychological well-being.	Group gardening settings to support social engagement.	Gooood (Design media platform) project page (full URL listed in References) (44)
C10	0.379	Accessible planting structures	Improves upper limb strength, physical coordination, and endurance.	Accessible-height raised planters for wheelchair-friendly gardening.	Gooood (Design media platform) project page (full URL listed in References) (44)

This table provides an author-prepared textual and structured summary of case-related design features. For complete visual materials and source details (including the full URL and access date), please refer to the corresponding entry in the reference list (Gooood project page).

At the theoretical level, horticultural activities are not simply a form of individual Physical Activity and Exercise, but also encompass processes such as learning about planting, multi-person collaboration, and the sharing of experience. In the course of participating in gardening, older adults can obtain emotional support, practical assistance, and physical help from others, which constitutes a concrete manifestation of the Social Support mechanism emphasized in Stress Reduction Theory; the same is true for other forms of social activities. By providing gardening activity spaces together with a variety of social settings, this design case meets older adults’ combined needs for socializing, interaction, and exercise. From the perspective of Attention Restoration Theory, it also reflects a high level of Compatibility between the environment and older adults’ behavioral goals and capabilities.

It should also be noted that, although the Longfor Yinian Apartment Therapeutic Garden performs strongly in Activity Accessibility (B6), Spatial Adaptability (B5), and Social Facilitation (B4), it does not hold a clear advantage in dimensions such as Natural Attractiveness (B1) and Environmental Tranquility (B2). Overall, this case places greater emphasis on creating spatial functionality and safety, while paying relatively insufficient attention to the naturalness and tranquil atmosphere that therapeutic landscapes seek to promote. As a rooftop garden, it is subject to inherent limitations in planting depth, spatial extensibility, and the creation of natural landscape features, which to some extent also reflect the structural constraints commonly faced by community green spaces in high-density urban environments. Future work should, while continuing to sustain strong performance in Activity Accessibility, Spatial Adaptability, and Social Facilitation, incorporate techniques such as vertical greening and soundscape optimization to further enhance the expression of dimensions related to Natural Attractiveness and Environmental Tranquility. Overall, the Longfor Yinian Apartment Therapeutic Garden, on the one hand, confirms the effectiveness of the evaluation framework developed in this study for identifying advantages in functional performance and safety; on the other hand, it reveals shortcomings in the creation of natural restorative environments in this type of community therapeutic garden, thereby providing useful directions for design optimization in future similar projects.

### 5.2 Design analysis of the Elizabeth Nona Evans therapeutic garden

The Elizabeth & Nona Evans therapeutic garden has an overall score of 0.354, ranking second among the three cases. Its main strengths are concentrated in dimensions such as Natural Attractiveness (B1), Exploration and Interest (B3), and Environmental Tranquility (B2), and it also shows certain advantages in Social Facilitation (B4). Overall, this case performs outstandingly in the creation of a natural environmental atmosphere, sensory experience, and aesthetic quality, while also accommodating certain functional needs. However, in the AHP weighting results of this study, the evaluation dimensions related to aesthetics and sensory experience carry relatively lower weights, so although this case has clear advantages in these aspects, it does not achieve an absolutely leading position in terms of the overall composite score. This divergence indirectly corroborates the value orientation of the present evaluation framework, which prioritizes functionality and safety. Nevertheless, this design case remains highly notable in its performance regarding natural landscape expression and its integration with functional design, providing a representative practical example for therapeutic landscapes.

Within the dimension of Natural Attractiveness (B1), the Elizabeth & Nona Evans Therapeutic Garden achieves its highest score in C2 Seasonal Landscape Variation (0.573), followed by C3 Sensory Appeal of the Landscape (0.533) (Table 13). Human beings’ innate biophilic tendencies make natural environments inherently attractive. In the Contemplation Garden within the Elizabeth & Nona Evans Therapeutic Garden, plants selected for their four-season interest are combined with carefully orchestrated color and fragrance compositions of flowers, as well as pathways and stone elements made from natural materials, to create a rich, multi-sensory and multi-layered seasonal landscape with strong sensory appeal. This environment provides older adults with continuous visual and olfactory stimulation and promotes positive psychological enjoyment. Existing studies have shown that seasonal variations in color, natural soundscapes (such as birdsong, water sounds, and wind), and floral scents can significantly alleviate stress and anxiety, improve blood pressure and heart rate, and promote autonomic nervous system balance (76–78). This case employs continuous “natural cues” and “gentle stimuli” to provide older adults with a stable and mild nature-based intervention setting, in which targeted interventions help users regulate their emotions and foster psychological enjoyment. In addition, the horticultural therapy area is planned as a sunny, open, and color-rich activity space. The planting areas are designed in a more natural manner rather than in the form of fixed planting beds and are highly adaptable, enabling both standing users and wheelchair users to experience the colors and fragrances of the plants at an appropriate visual height.

**Table 13:** The design of "natural attractiveness" in Elizabeth Nona Evans therapeutic garden.

**Natural attractiveness B1 sub-criteria**	**Weight**	**Design strategies**	**Health benefits**	**Case description**	**Source for original visuals (website)**
C2	0.573	Contemplation garden with green-dominant palette	Provides strong visual appeal, enhances psychological well-being, regulates mood, and promotes relaxation.	Green planting and lawn views for calm contemplation	ASLA awards page (full URL listed in References) (43)
C2	0.573	Nature-based pathway	Improves spatial accessibility while offering a smooth and immersive visual experience.	Nature-integrated paths for smooth access and immersive views	ASLA awards page (full URL listed in References) (43)
C3	0.533	Horticultural therapy	Delivers sensory stimulation and therapeutic experience; appropriate plant/facility height ensures privacy without complete isolation, enhancing a sense of safety.	Accessible gardening areas for sensory and therapeutic activity.	ASLA awards page (full URL listed in References) (43)

This table provides an author-prepared textual and structured summary of case-related design features. For complete visual materials and source details (including the full URL and access date), please refer to the corresponding entry in the reference list (ASLA awards page).

Compared with the more function-oriented Longfor Yinian Apartment Therapeutic Garden, the Elizabeth & Nona Evans Therapeutic Garden places greater emphasis on creating an attractive natural environment through planting design, water features, and the use of natural materials. The entire garden forms a composite sensory environment characterized by seasonal colors, natural soundscapes, floral fragrances, and tactile experiences, providing users with continuous “nature-based interventions” that indirectly support improvements in older adults’ emotional and cognitive states and enhance their overall psychological health. This process constitutes a concrete manifestation of the Nature-Based Interventions mechanism articulated in Stress Reduction Theory. At the same time, the multi-layered and detail-rich landscape scenes provide users with viewing objects and places to pause that are sufficiently engaging to hold their attention, so that people are unconsciously drawn to the environment and willing to linger and gaze. This is highly consistent with the fascination-type environmental characteristics emphasized in Attention Restoration Theory.

In the dimension of Exploration and Interest (B3), the Elizabeth & Nona Evans Therapeutic Garden received high scores for Spatial Discoverability (C6, 0.567), Dynamic Landscapes (C7, 0.516), and Mystery and Layering (C8, 0.574). These results indicate that the case features a richly layered landscape capable of stimulating older adults’ exploratory interest and enhancing their initiative to engage in outdoor activities (Table 14). Existing studies have shown that interacting with natural environments through multi-sensory pathways such as olfactory and tactile perception can help reduce agitated behaviors and improve sleep quality and emotional states (79). At the same time, environments with rich detail and layered characteristics are more likely to elicit interactive and exploratory behaviors, thereby promoting attention restoration and reducing psychological stress (80). In design terms, this case introduces aromatic plants and vertical greening installations along the walls to encourage older adults to interact with the varied textures and changing scents of the stone surfaces, thereby enhancing the spatial explorability of the space and exercising the tactile sensitivity of their hands. In terms of dynamic landscapes, the garden creates rich waterscapes through waterfalls, pools, and streams flowing over moss-covered stone walls. These water features not only provide visually dynamic effects but also enhance auditory experience through the sound of water, and to some extent mitigate the heat island effect and environmental noise. By stimulating a bodily sense of connection with nature, they can significantly reduce stress, enhance mental pleasure, and offer emotional comfort (81). In terms of creating a sense of mystery and spatial layering, the Contemplation Garden in the Elizabeth & Nona Evans therapeutic garden uses the enclosure formed by low retaining walls and tall stone walls to define a relatively private interior space. Combined with multi-level spatial partitions and deliberate visual screening, this configuration creates a setting that feels both safe and subtly “semi-hidden,” encouraging visitors to explore gradually under the combined influence of tactile, olfactory, and auditory stimuli. The stone walls themselves function not only as physical boundaries but also as integral components of the exploratory landscape. By using locally sourced stone with varied surface textures and allowing plants to cascade naturally from wall niches, the garden provides older adults with direct opportunities to touch the stone, brush against the foliage, and smell the flowers. Such intentional tactile stimulation helps activate relevant brain regions, thereby contributing to the delay of cognitive decline and alleviation of cognitive impairment. Moreover, it has been shown to reduce psychological stress, ease anxiety and depressive symptoms, and promote overall mental well-being.

**Table 14:** The design of "exploration and interest" in Elizabeth Nona Evans therapeutic garden.

**Exploration and interest B3 sub-criteria**	**Weight**	**Design measures**	**Health benefits**	**Case description**	**Source for original visuals (website)**
C6	0.567	Aromatic plants and vertical greenery	Enhances tactile sensitivity in the hands.	Aromatic and vertical planting for sensory exploration.	ASLA awards page (full URL listed in References) (43)
C7	0.516	Dynamic landscapes created with water features	Reduces stress, enhances emotional well-being, and provides psychological comfort.	Water features for dynamic interest and calming effects.	ASLA awards page (full URL listed in References) (43)
C8	0.574	Low retaining walls for spatial layering	Supports attention restoration; enhances privacy and sense of safety.	Low walls and layered planting for enclosure and variety.	ASLA awards page (full URL listed in References) (43)
C8	0.574	Rich tactile environmental textures	Delays cognitive decline; reduces psychological stress, alleviates anxiety and negative emotions, and promotes mental health.	Varied textures along paths for tactile engagement.	ASLA awards page (full URL listed in References) (43)

This table provides an author-prepared textual and structured summary of case-related design features. For complete visual materials and source details (including the full URL and access date), please refer to the corresponding entry in the reference list (ASLA awards page).

Overall, within the evaluation framework of this study, this case exhibits a high degree of design differentiation on the dimensions related to Exploration and Interest, and aligns closely with the theoretical underpinnings of therapeutic landscapes. On the one hand, elements such as waterfalls, pools, moss-covered stone walls, and aromatic plants jointly constitute continuous natural stimuli that act on users through multiple sensory channels, representing a concrete manifestation of the Nature-Based Interventions mechanism in Stress Reduction Theory. On the other hand, the layered spatial sequence organized by stone walls, planting, and waterscapes not only creates multiple scenes imbued with a sense of mystery, thereby reinforcing the environment’s fascination, but also provides older adults with a clearly bounded overall extent within which they can freely explore and linger. This is consistent with the environmental characteristics emphasized in Attention Restoration Theory, and thus offers strong support for the restoration of older adults’ cognitive functioning and the regulation of their emotional states.

In terms of Environmental Tranquility (B2), the garden also demonstrates clear advantages, particularly in C5 Tranquil Areas for a Sense of Privacy, where it performs especially well with a score of 0.507. Through a comprehensive strategy that integrates physical spatial separation, soundscape design, and planting arrangement, the design effectively creates a calm and tranquil spatial atmosphere (Table 15). Relevant studies have shown that small-scale, enclosed green spaces are conducive to cognitive restoration, emotional improvement, and psychological relaxation (82). At the same time, vegetative enclosure, natural water features, and shading facilities collectively enhance thermal comfort and perceived safety in the environment (83). Exposure to natural sounds (such as flowing water and wind rustling through leaves) can significantly reduce heart rate and blood pressure at the physiological level, while at the psychological level it helps to alleviate anxiety, restore attention, and, by masking external noise, enhance the perceived tranquility of the environment (84). These findings suggest that a diverse set of environmental elements centered on planting and waterscapes constitutes a critical foundation for constructing therapeutic landscapes with high levels of tranquility and restorative capacity. In terms of concrete design expression, this case implements the requirements of the Environmental Tranquility (B2) dimension through multiple design strategies. First, in terms of spatial form, the design incorporates a viewing platform enclosed by layered planting, waterscapes, and locally sourced stone, creating a semi-private resting space of appropriate scale with a strong sense of enclosure. This space not only offers good outward views but also attenuates external disturbances, thereby enhancing users’ sense of immersion. At the garden-wide planting level, a combination of dense trees and low shrubs is employed, which not only reinforces visual layering but also strengthens the sense of enclosure and perceived safety. Second, in terms of soundscape design, the garden makes extensive use of small-scale waterscapes such as waterfalls and streams, and replaces artificial background music with the sounds of gently flowing water and wind rustling through the leaves, thereby creating an acoustic environment dominated by natural sound sources. By masking external noise and improving the local microclimate, this soundscape enables visitors to experience greater comfort and relaxation in terms of blood pressure, emotional state, and cognitive functioning (85). Third, in terms of barrier-free and sensory-friendly design, the scheme incorporates braille poetry on the back of the railings, guiding people with visual impairments to engage more deeply with the environment through touch and imaginative reading of the text. In combination with the subtle changes in temperature and humidity brought about by the flowing water, this enables visitors with different sensory abilities to experience a sense of peace and enjoyment in the space.

**Table 15:** The design of "environmental tranquility" in Elizabeth Nona Evans therapeutic garden.

**Environmental tranquility B2 sub-criteria**	**Weight**	**Design measures**	**Health benefits**	**Case description**	**Source for original visuals (website)**
C5	0.507	Spatial separation	Enhances privacy and tranquility, promoting attention restoration.	Planting buffers for privacy and quiet.	ASLA awards page (full URL listed in References) (43)
C5	0.507	Braille-integrated handrails	Alleviates arthritis symptoms. Enhances a sense of safety and engagement, relieving stress and elevating mood.	Tactile (Braille) handrails for safe navigation.	ASLA awards page (full URL listed in References) (43)
C5	0.507	Soundscape design	Blocks external noise, soothes emotions, improves blood pressure, and enhances cognitive function.	Layout and vegetation to reduce noise.	ASLA awards page (full URL listed in References) (43)
C5	0.507	Multilayered planting	Reduces external disturbances, improves visual perception, and uplifts mental well-being.	Layered planting to buffer disturbance.	ASLA awards page (full URL listed in References) (43)

This table provides an author-prepared textual and structured summary of case-related design features. For complete visual materials and source details (including the full URL and access date), please refer to the corresponding entry in the reference list (ASLA awards page).

From a theoretical perspective, the performance of this design case in the Environmental Tranquility (B2) dimension is likewise consistent with Stress Reduction Theory (SRT) and Attention Restoration Theory (ART). Through the combined visual, auditory, olfactory, and tactile effects of planting, waterscapes, and enclosing structures, the garden, on the one hand, continuously exerts nature-based interventions on users via natural elements, helping to alleviate stress. On the other hand, by attenuating external noise and disturbances, it enables individuals to experience a sense of being away from everyday stressors in both psychological and spatial perception, thereby providing a quiet space for rest and restoration. Within the evaluation framework of this study, this case illustrates that the sub-indicators related to Environmental Tranquility have considerable explanatory power for identifying therapeutic landscapes that center on tranquil experiences, and that they can function in a complementary way to the dimensions of Natural Attractiveness and Exploration and Interest.

It should be emphasized that, although the Elizabeth & Nona Evans Therapeutic Garden performs prominently in the dimensions of Natural Attractiveness, Exploration and Interest, and Environmental Tranquility, it does not hold a particular advantage in more functional dimensions such as Activity Accessibility (B6) and Spatial Compatibility (B5). For example, in terms of barrier-free facilities, continuous pathways, and the organization of exercise spaces, this case is primarily oriented toward integrating the natural environment into the therapeutic garden as its core design objective, while showing certain limitations in systematically meeting the needs of older adults for walking training, rehabilitative exercise, and high-frequency everyday activities. This finding suggests that, even for traditionally conceived high-quality therapeutic gardens that perform strongly in terms of naturalness and restorative qualities, the absence of spatial configurations that closely align with older adults’ everyday behavior patterns and functional needs may still place them at a relative disadvantage in the overall evaluation.

### 5.3 The design analysis of the sun city Kobe senior living apartment in Japan

Japan’s Sun City Kobe Retirement Residence achieved an overall score of 0.209, ranking last among the three design cases. However, it still performs well in the dimensions of Environmental Tranquility (B2) and Natural Attractiveness (B1). Overall, this case shows certain advantages in the creation of a natural environmental atmosphere and sensory experience, whereas its performance is relatively weak in more function-oriented community therapeutic spaces, such as Activity Accessibility (B6) and Spatial Compatibility (B5). In parallel, the evaluation framework developed in this study assigns greater weight to environmental characteristics related to functionality and safety, whereas the dimensions associated with sensory experience and natural ambience receive relatively lower weights. Consequently, even though this case has clear advantages in terms of naturalness and sensory qualities, it is still difficult for it to attain a high position in the overall evaluation ranking. Nevertheless, the design strategies employed in Japan’s Sun City Kobe Retirement Residence for enhancing natural attractiveness and cultivating a tranquil atmosphere still provide valuable insights for community therapeutic landscape practice for older adults.

In terms of Environmental Tranquility (B2) dimension, both C4 Spatial Design for Noise Isolation (0.3505) and C5 Tranquil Areas for a Sense of Privacy (0.3357) perform well (Table 16). The landscape design concept of Japan’s Sun City Kobe Retirement Residence emphasizes the integration of the urban and natural environment. Drawing on the natural landscape of Mount Rokko, the design uses the surrounding natural environment, rather than simply buildings or walls, as spatial boundaries. This form of physical separation achieved through natural settings generates a sense of being away, which can more effectively alleviate psychological stress in older adults (86). At the same time, a diverse selection of seasonal vegetation is employed to create visual separation, which contributes to reducing stress, anxiety, and depressive symptoms while enhancing emotional resilience and subjective well-being (87). The verdant planting environment serves to augment the site’s ecological value by establishing conducive habitats for wildlife, thereby enabling senior residents to experience the vibrancy of nature within the garden. This interactive setting—shared by humans, animals, and plants—has been demonstrated to markedly alleviate feelings of loneliness, foster a sense of security and emotional connection, and effectively stimulate social engagement, cognitive restoration, and verbal expression (88). Additionally, existing studies have shown that even when individuals remain indoors, simply viewing outdoor natural scenery through a window can, to some extent, promote physiological restoration and emotional regulation (89). In this design case, private indoor spaces are connected to the courtyard landscape through large, transparent floor-to-ceiling windows, allowing residents to appreciate the changing natural scenery across the seasons even under adverse weather conditions. This visual connection enhances the comfort of the residential environment and overall life satisfaction, while also promoting emotional stability and psychological well-being.

**Table 16:** The design of "environmental tranquility" in Japan sun city kobe older adults apartment.

**Environmental tranquility B2 sub-criteria**	**Weight**	**Design measures**	**Health benefits**	**Case description**	**Source for original visuals (website)**
C4	0.3505	Spatial separation through geographic environment	Relieves stress.	Courtyard views and setbacks for calm.	Mooool project page; Archina project page (full URLs listed in References) (45, 46)
C4	0.3505	Spatial buffering via plant arrangements	Reduces anxiety and depressive symptoms; enhances emotional resilience and subjective well-being.	Planting buffers for quiet separation.	Mooool project page; Archina project page (full URLs listed in References) (45, 46)
C5	0.3357	Habitat creation for wildlife	Reduces loneliness, fosters security and emotional bonds, and supports social and cognitive engagement.	Wildlife habitats for nature contact.	Mooool project page; Archina project page (full URLs listed in References) (45, 46)
C5	0.3357	Residential spaces connected to outdoor landscapes	Facilitates emotional stability and enhances psychological well-being.	Semi-outdoor seating with garden views.	Mooool project page; Archina project page (full URLs listed in References) (45, 46)

This table provides an author-prepared textual and structured summary of case-related design features. For complete visual materials and source details (including full URLs and access dates), please refer to the corresponding entries in the reference list (Mooool and Archina project pages).

At the theoretical level, this design exhibits a high degree of consistency with the nature-based interventions mechanism in Stress Reduction Theory (SRT) and the being away environmental characteristic in Attention Restoration Theory (ART). On the one hand, by combining diverse natural elements such as animals, plants, mountain landforms, and waterscapes, it creates rich sensory experiences and interactive situations, enabling users, under continuous exposure to natural stimuli, to alleviate anxiety, improve their emotional state, and enhance cognitive functioning. On the other hand, the separation of natural spaces from the surrounding urban environment, together with the spatial separation between buildings and landscapes and between different landscape areas, creates a physical sense of being away, enabling older adults to temporarily detach from everyday stressors and thereby facilitating the restoration of attention and emotional states. Moreover, because Environmental Tranquility (B2) does not carry a high weight within the evaluation framework, this design, although performing relatively well in this regard, does not achieve an ideal overall rating, and there remains substantial room for improvement in further reducing noise disturbance and strengthening the sense of tranquility in the environmental atmosphere.

In terms of Natural Attractiveness (B1), the indicator C1 Aesthetic Appeal and Attractiveness (0.3050) performs relatively well. An increasing body of research has demonstrated that artistic interventions play an important role in disease prevention, health promotion, and adjunctive treatment (90). Accordingly, the landscape design of Sun City Kobe Retirement Residence draws inspiration from art festivals, incorporating modern sculptures, small waterfalls, and streamlined architectural forms to create spaces that are rich in artistic ambience yet quiet and elegant (Table 17). The courtyard garden features white “island” sculptures that gently divide the waterfall streams, thereby creating a layered and visually dynamic waterscape. These sculptural forms reflect aspects of local culture, enhancing residents’ sense of belonging and encouraging social engagement (91). Relevant studies have indicated that moderate waterscapes and natural sound environments can produce a calming effect, stimulating parasympathetic nervous system activity and helping to alleviate tension, anxiety, and related emotional stress, thereby promoting restorative regulation of the nervous system (92). In this design case, the fountain functions as a focal, point-like landscape element strategically interspersed between buildings to create diverse water-environment experiences. It enhances the visual appeal of the landscape while improving sensory interaction and overall environmental quality. At the rear, a white viewing platform, conceived with the image of a “floating pier,” is suspended above the water, creating a formal linkage between the architecture and the surrounding landscape. By combining waterscapes, sculptures, and platforms, this design case reinforces the environmental attractiveness at both visual and behavioral levels, providing a spatial setting for informal encounters and social interaction among older adults and, to some extent, supporting a more integrated experience of social participation.

**Table 17:** The design of "natural attractiveness" in Japan sun city kobe older adults apartment.

**Natural attractiveness B1 sub-criteria**	**Weight**	**Design measures**	**Health benefits**	**Case description**	**Source for original visuals (website)**
C1	0.305	“Island”-shaped sculpture	Enhances sense of belonging and promotes social engagement.	Landmark sculpture for place identity.	Mooool project page; Archina project page (full URLs listed in References) (45, 46)
C1	0.305	Water features	Provides calming effects, activates the nervous system, and alleviates tension and anxiety.	Water features for calming effects	Mooool project page; Archina project page (full URLs listed in References) (45, 46)
C1	0.305	Viewing platform	Strengthens social participation and interpersonal interaction.	Viewing platform for pause and social interaction.	Mooool project page; Archina project page (full URLs listed in References) (45, 46)

This table provides an author-prepared textual and structured summary of case-related design features. For complete visual materials and source details (including full URLs and access dates), please refer to the corresponding entries in the reference list (Mooool and Archina project pages).

From the perspective of Stress Reduction Theory (SRT) and Attention Restoration Theory (ART), this design case primarily employs artfully designed forms of nature-based intervention, creating waterscapes and sculptures that exhibit the environmental characteristic of *fascination*. These features provide older adults with engaging objects of contemplation and places to linger, thereby, to some extent, facilitating short-term emotional regulation and the restoration of attention.

Although Japan’s Sun City Kobe Retirement Residence performs relatively strongly in Environmental Tranquility (B2) and Natural Attractiveness (B1), its comparatively weak performance in functional dimensions such as Activity Accessibility (B6), Spatial Compatibility (B5), and Social Facilitation (B4) results in a weaker overall evaluation. In design practice targeting community-based older adult care and rehabilitation needs, if functional spaces that support older adults’ everyday behavior patterns are lacking, and the therapeutic environment is constructed primarily around landscape ambience by relying solely on the potential fascination and being away qualities of natural scenery, it is difficult for such a case to attain an advantageous position in comprehensive evaluations. This finding indicates that, in the context of high-density urban environments, the design of community therapeutic landscapes for older adults needs not only to focus on cultivating landscape ambience, but also to systematically incorporate barrier-free circulation, rehabilitation training spaces, and easily accessible social spaces. In this way, the natural restorative qualities of therapeutic landscape spaces can be expressed while more effectively meeting the practical needs of older adults.

## 6 Limitations and future directions

This study evaluated and analyzed three design cases to clarify the strengths and weaknesses of current therapeutic landscape design for older adults. However, there are still certain limitations in terms of data availability, research methods, and results. In terms of data acquisition, first, due to constraints on physical access to the sites, the experts’ evaluations of the design cases relied primarily on publicly available online resources rather than on-site observations. This reliance on secondary materials may, to some extent, compromise the accuracy of the evaluation. Second, during the expert scoring process, the evaluations relied largely on the experts’ own knowledge, and the limited photographic information made it difficult to capture spatial perceptions such as soundscape and microclimate, which may have introduced some bias into certain scoring results. Therefore, future studies should incorporate field investigations, including environmental measurements and on-site assessments of the design cases, in order to improve the reliability of the evaluation scores. In terms of research methods, the Analytic Hierarchy Process (AHP) theoretically assumes that evaluation criteria at the same level are independent of one another, whereas, in practice, the evaluation framework for therapeutic landscapes involves design dimensions that are often intrinsically interrelated within actual spatial configurations. The weights derived in this study should therefore be interpreted as an ordering of relative importance based on the current theoretical framework and expert judgments, rather than as precise estimates obtained under a strict assumption of statistical independence. Future research could adopt the Analytic Network Process (ANP) or structural equation modeling (SEM) to further test and refine the interrelationships among the dimensions. In terms of results, because dedicated therapeutic landscape projects serving older adults are relatively limited in number, the three design cases examined in this study differ to some extent in scale, regional environment, cultural background, and functional facilities. These differences may have introduced a certain degree of bias into the expert evaluation results. Moreover, because this study primarily evaluates, in a theoretical sense, whether landscape spaces that take older adults as their main service group possess the characteristics of therapeutic landscapes, it has, to some extent, reduced the emphasis on older users’ own evaluations of and feedback on the environment. Future research should also conduct interviews, questionnaire surveys, and physical health assessments with older adults who have used specific therapeutic landscape spaces, in order to explore the target population’s actual evaluations of, and feedback on, these places. This would help to remedy the one-sidedness of relying solely on expert scoring as the evaluation standard and, by integrating design theory, user feedback, and therapeutic effects, provide clear guidance for the further development of therapeutic landscape design.

## 7 Conclusion

Grounded in therapeutic landscape theory, this study integrates Stress Reduction Theory (SRT) and Attention Restoration Theory (ART) to distill a set of therapeutic landscape characteristics centered on Natural Attractiveness, Environmental Tranquility, Exploration and Interest, Social Facilitation, Spatial Adaptability, and Activity Accessibility, and to construct an evaluation framework for community therapeutic landscapes for older adults. The establishment of this evaluation framework represents a further step in theory-to-method development, translating abstract theoretical concepts into an operational system of evaluative indicators. At the same time, the study introduces the Analytic Hierarchy Process (AHP), inviting 11 experts in the field to rate the therapeutic landscape characteristic dimensions and the selected design cases. At the theoretical level, this clarifies the weights of each therapeutic landscape dimension within the evaluation framework, while at the empirical level, it verifies the current practice of applying Stress Reduction Theory (SRT) and Attention Restoration Theory (ART) in the age-friendly design of community green spaces, thereby further clarifying the development from theory to practice.

Through ranking the weights of the therapeutic landscape characteristic dimensions, this study clearly demonstrates the relative importance and discriminative power of each theoretical dimension in the community context. It further indicates that functional spaces represented by Activity Accessibility, Spatial Adaptability, and Social Facilitation exhibit a pronounced “threshold effect” in current practice of community therapeutic landscape design for older adults. This suggests that age-friendly design of community therapeutic landscapes should take the fulfillment of safety and functional requirements as a fundamental precondition. By improving barrier-free facilities, lighting and wayfinding systems, and walkable, accessible environments, the comfort and convenience of these spaces can be enhanced. At the same time, the incorporation of horticultural therapy and appropriately designed social spaces, with the aim of fostering social support, can help ensure that community therapeutic landscapes truly function as spatial carriers of emotional connection among community residents. Based on the evaluation and analysis of the three typical design cases using the therapeutic landscape characteristic dimension framework, this study clarifies the differences in implementation pathways of therapeutic landscapes within current community age-friendly design practice. The Longfor Yinian Apartment Therapeutic Garden is predominantly function-oriented, constructing a safe, controllable, and adaptable outdoor environment for exercise and social interaction through continuous pathways, dual-level handrails, and multi-layered fitness and exercise spaces. The Elizabeth & Nona Evans Restorative Garden emphasizes the creation of seasonally changing, multi-sensory, and highly explorable spaces through the natural environment, providing a referential pathway for enhancing naturalness and tranquility in community therapeutic landscapes. The Sun City Kobe Retirement Residence in Japan demonstrates, in the context of mountainous terrain and a high-density urban setting, the potential to integrate natural geographical conditions with artistic and cultural landscape elements to achieve a synthesis of environmental tranquility and aesthetics. Each of the three design cases has its own strengths, not only offering practical references for the design expression of each therapeutic landscape characteristic dimension, but also indicating directions for further refinement of future design practice.

Overall, at the theoretical level, this study enriches the theoretical toolkit for research on community therapeutic landscapes for older adults and age-friendly environments by constructing an evaluation framework and weighting system for therapeutic landscape characteristic dimensions. At the practical level, the weights derived from the evaluation framework, together with the evaluations of the design cases, provide quantitative evidence for community regeneration, the development of supporting green spaces for eldercare facilities, and the formulation of relevant standards and design guidelines. At the same time, under conditions of limited resources, they offer designers and decision-making bodies more targeted strategies for spatial configuration and optimization.

## Data Availability

The original contributions presented in the study are included in the article/Supplementary material, further inquiries can be directed to the corresponding author.

## Footnotes

1
https://web.archive.org/web/20241111021149/https://www.asla.org/awards/2006/06winners/294.html

2
https://www.gooood.cn/caring-landscape-friendly-garden-longfor-yinian-apartment-rehabilitation-garden-chongqing-china-gvl.htm

3
https://mooool.com/sun-city-kobe-tower-by-richard-beard-architects.html?utm_source=chatgpt.com

4
http://www.archina.com/index.php?g=works&m=index&a=show&id=5650

## References

[1] United Nations, Department of Economic and Social Affairs, Population Division. (2024). World population prospects 2024: Summary of results [internet]. New York: United Nations. Available online at: https://population.un.org/wpp/assets/Files/WPP2024_Summary-of-Results.pdf (Accessed March 18, 2025).

[2] Nicholson K Liu W Fitzpatrick D Hardacre KA Roberts S Salerno J . Prevalence of multimorbidity and polypharmacy among adults and older adults: a systematic review. Lancet Healthy Longev. (2024) 5:e287–96. doi: 10.1016/S2666-7568(24)00007-2, 38452787

[3] Pais R Ruano L Carvalho OP Barros H. Global cognitive impairment prevalence and incidence in community-dwelling older adults—a systematic review. Geriatrics. (2020) 5:84. doi: 10.3390/geriatrics5040084, 33121002 PMC7709591

[4] World Health Organization. (2023). Progress report on the United Nations decade of healthy ageing, 2021–2023. Geneva: WHO. Available online at: https://www.who.int/publications/m/item/decade-of-healthy-ageing-plan-of-action (Accessed April 12, 2025).

[5] Jørgensen KE. What is community? In: What is international relations? Bristol: Bristol University Press (2021). 106–20.

[6] Chavis DM Lee K. What is community anyway? Stanf Soc Innov Rev. (online). (2015). doi: 10.48558/EJJ2-JJ82

[7] Marques B McIntosh J Kershaw C. Therapeutic environments as a catalyst for health, well-being and social equity. Landsc Res. (2021) 46:766–81. doi: 10.1080/01426397.2021.1906851

[8] Han Y Liang Y. Scientific knowledge map study of therapeutic landscapes and community open spaces: visual analysis with CiteSpace. Sustainability. (2023) 15:15066. doi: 10.3390/su152015066

[9] Ulrich RS. View through a window may influence recovery from surgery. Science. (1984) 224:420–1. doi: 10.1126/science.6143402, 6143402

[10] Kaplan R Kaplan S. The experience of nature: A psychological perspective. Cambridge: Cambridge University Press (1989).

[11] Gesler WM. Therapeutic landscapes: medical issues in light of the new cultural geography. Soc Sci Med. (1992) 34:735–46. doi: 10.1016/0277-9536(92)90360-3, 1376497

[12] Williams A. Therapeutic landscapes in holistic medicine. Soc Sci Med. (1998) 46:1193–203. doi: 10.1016/S0277-9536(97)10048-X, 9572609

[13] Conradson D. Landscape, care and the relational self: therapeutic encounters in rural England. Health Place. (2005) 11:337–48. doi: 10.1016/j.healthplace.2005.02.004, 15886142

[14] Lyshol H Johansen R. The association of access to green space with low mental distress and general health in older adults: a cross-sectional study. BMC Geriatr. (2024) 24:329. doi: 10.1186/s12877-024-04738-3, 38600442 PMC11007904

[15] Whear R Coon JT Bethel A Abbott R Stein K Garside R. What is the impact of using outdoor spaces such as gardens on the physical and mental well-being of those with dementia? A systematic review of quantitative and qualitative evidence. J Am Med Dir Assoc. (2014) 15:697–705. doi: 10.1016/j.jamda.2014.05.013, 25037168

[16] Meneghetti C Murroni V Borella E Melendugno A Carbone E Goldin G . Psychological impacts of intervention to improve a therapeutic garden for older adults with dementia: a case study conducted at a care facility. Front Psychol. (2023) 14:1183934. doi: 10.3389/fpsyt.2023.1183934, 37234215 PMC10206005

[17] Pimentel HCB de Lima APM Latawiec AE. Recommendations for implementing therapeutic gardens to enhance human well-being. Sustainability. (2024) 16:9502. doi: 10.3390/su16219502

[18] Harries B Chalmin-Pui LS Gatersleben B Griffiths A Ratcliffe E. ‘Designing a wellbeing garden’: a systematic review of design recommendations. Des Health. (2023) 7:180–201. doi: 10.1080/24735132.2023.2215915

[19] Ulrich RS Simons RF Losito BD Fiorito E Miles MA Zelson M. Stress recovery during exposure to natural and urban environments. J Environ Psychol. (1991) 11:201–30. doi: 10.1016/S0272-4944(05)80184-7

[20] Gesler WM. Therapeutic landscapes: theory and a case study of Epidauros, Greece. Environ Plan D Soc Space. (1993) 11:171–89. doi: 10.1068/d110171

[21] Williams A. Therapeutic landscapes: The dynamic between place and wellness. Lanham, New York, Oxford: University Press of America, Inc. (1999).

[22] Ulrich RS. Aesthetic and affective response to natural environment In: Altman I Wohlwill JF, editors. Behavior and the natural environment. New York: Springer (1983). 85–125. doi: 10.1007/978-1-4613-3539-9_4

[23] Ulrich RS. Health benefits of gardens in hospitals In: Plants for people: International Exhibition Floriade. The Netherlands: Flower Council of Holland (2002)

[24] Marcus CC Sachs NA. Therapeutic landscapes: An evidence-based approach to designing healing gardens and restorative outdoor spaces. Hoboken (NJ): John Wiley & Sons (2013). 10.1177/193758671400700413 25303434

[25] Ulrich RS. Biophilic theory and research for healthcare design In: Kellert SR Heerwagen JH Mador ML, editors. Biophilic design: The theory, science, and practice of bringing buildings to life. Hoboken (NJ): Wiley (2008). 87–106.

[26] Ulrich RS Berry LL Quan X Parish JT. A conceptual framework for the domain of evidence-based design. HERD. (2010) 4:95–114. doi: 10.1177/193758671000400107, 21162431

[27] Kaplan R Kaplan S. The experience of nature: A psychological perspective. Cambridge: Cambridge University Press (1989).

[28] Kaplan S. The restorative benefits of nature: toward an integrative framework. J Environ Psychol. (1995) 15:169–82. doi: 10.1016/0272-4944(95)90001-2

[29] Kaplan R. The nature of the view from home: psychological benefits. Environ Behav. (2001) 33:507–42. doi: 10.1177/00139160121973115

[30] Basu A Duvall J Kaplan R. Attention restoration theory: exploring the role of soft fascination and mental bandwidth. Environ Behav. (2019) 51:1055–81. doi: 10.1177/0013916518774400, 41405032

[31] Hartig T. Restoration in nature: beyond the conventional narrative In: Schutte AR Torquati JC Stevens JR, editors. Nature and psychology: Biological, cognitive, developmental, and social pathways to well-being. Cham: Springer International Publishing (2021). 89–151.

[32] Van den Berg AE Hartig T Staats H. Preference for nature in urbanized societies: stress, restoration, and the pursuit of sustainability. J Soc Issues. (2007) 63:79–96. doi: 10.1111/j.1540-4560.2007.00497.x

[33] Berman MG Jonides J Kaplan S. The cognitive benefits of interacting with nature. Psychol Sci. (2008) 19:1207–12. doi: 10.1111/j.1467-9280.2008.02225.x, 19121124

[34] Hartig T Evans GW Jamner LD Davis DS Gärling T. Tracking restoration in natural and urban field settings. J Environ Psychol. (2003) 23:109–23. doi: 10.1016/S0272-4944(02)00109-3

[35] Johnsen SÅK Brown MK Rydstedt LW. Restorative experiences across seasons? Effects of outdoor walking and relaxation exercise during lunch breaks in summer and winter. Landsc Res. (2022) 47:664–78. doi: 10.1080/01426397.2022.2063268

[36] Berto R. Exposure to restorative environments helps restore attentional capacity. J Environ Psychol. (2005) 25:249–59. doi: 10.1016/j.jenvp.2005.07.001

[37] Grave AJJ Neven L Mohammadi M. Reconsidering restorative environments for older adults: how the social context shapes restorative experiences. J Environ Psychol. (2025) 105:102636. doi: 10.1016/j.jenvp.2025.102636

[38] Bissell J Eyres J Gulwadi GB Harper E Kirk N Lee S . Environmental design research association In: Fernando NA Barker GA, editors. Proceedings of the 46th Annual Conference of the Environmental Design Research Association. Los Angeles, CA: Environmental Design Research Association (EDRA) (2015). 27–30.

[39] Roberts H van Lissa C Hagedoorn P Kellar I Helbich M. The effect of short-term exposure to the natural environment on depressive mood: a systematic review and meta-analysis. Environ Res. (2019) 177:108606. doi: 10.1016/j.envres.2019.108606, 31362251

[40] Han Y Choi KR Miao JJ. A study on the design framework for therapeutic green spaces for the elderly. J Korea Inst Spat Des. (2025) 20:419–32. doi: 10.35216/kisd.2025.20.3.419

[41] National Bureau of Statistics of China. (2024). 年国民经济和社会发展统计剬报 (解读). Available online at: https://www.stats.gov.cn/sj/sjjd/202501/t20250117_1958337.html?utm_source=chatgpt.com (Accessed March 25, 2025).

[42] Statistics Bureau of Japan. (2024). 我が国の人口推計(令和6年(2024年)10月1日現在)トピックス No.142 [Internet]. Available online at: https://www.stat.go.jp/data/topics/pdf/topics142.pdf?utm_source=chatgpt.com (Accessed April 8, 2025).

[43] American Society of Landscape Architects (ASLA). (2006). 2006 professional awards: Healing Garden, Cleveland Botanical Garden [Internet]. Available online at: https://web.archive.org/web/20241111021149/https://www.asla.org/awards/2006/06winners/294.html (Accessed March 22, 2025).

[44] gooood (2018). 关怀型景观:龙湖颐年剬寓康复花园, 重庆, 中国, GVL [Internet]. Available online at: https://www.gooood.cn/caring-landscape-friendly-garden-longfor-yinian-apartment-rehabilitation-garden-chongqing-china-gvl.htm (accessed March 27, 2025).

[45] mooool (2020). Sun City Kobe Tower by Richard Beard Architects [Internet]. Available online at: https://mooool.com/sun-city-kobe-tower-by-richard-beard-architects.html?utm_source=chatgpt.com (accessed April 5, 2025).

[46] SWA. (2020). Sun City Kobe Retirement Residence landscape design, Kobe, Japan [Internet]. Available online at: http://www.archina.com/index.php?g=works&m=index&a=show&id=5650 (accessed May 2025).

[47] Saaty TL. A scaling method for priorities in hierarchical structures. J Math Psychol. (1977) 15:234–81. doi: 10.1016/0022-2496(77)90033-5

[48] Saaty TL. The analytic hierarchy process (AHP). J Oper Res Soc. (1980) 41:1073–6. doi: 10.1057/jors.1980.178

[49] Bhushan N Rai K. The analytic hierarchy process In: Strategic decision making: Applying the analytic hierarchy process. London: Springer (2004). 11–21.

[50] Darko A Chan APC Ameyaw EE Owusu EK Pärn E Edwards DJ. Review of application of analytic hierarchy process (AHP) in construction. Int J Constr Manag. (2019) 19:436–52. doi: 10.1080/15623599.2018.1452098, 41307611

[51] Saaty TL. The analytic hierarchy and analytic network processes for the measurement of intangible criteria and for decision-making In: Figueira J Greco S Ehrgott M, editors. Multiple criteria decision analysis: State of the art surveys. New York, NY: Springer (2005). 345–405.

[52] Cheng Q Li Q. The application of grey statistical method and analytic hierarchy process in the evaluation of community park rehabilitation landscapes. Humanit Soc Sci Commun. (2025) 12:158. doi: 10.1057/s41599-024-04157-0

[53] Dong W Sun S Fu Y. Assessing urban community parks from an age-friendly perspective: a multi-criteria decision-making approach. Front Public Health. (2025) 13:1663359. doi: 10.3389/fpubh.2025.1663359, 41283038 PMC12634358

[54] Weng C-K Lai C-F Yeh W-C. Evaluation index for healing gardens in computer-aided design. Eng Proc. (2025) 98:17. doi: 10.3390/engproc2025098017

[55] Saaty TL. Decision making with the analytic hierarchy process. Int J Serv Sci. (2008) 1:83–98. doi: 10.1504/IJSSCI.2008.017590, 35009967

[56] Saaty TL. How to make a decision: the analytic hierarchy process. Eur J Oper Res. (1990) 48:9–26. doi: 10.1016/0377-2217(90)90057-I 11659401

[57] Saaty TL Vargas LG. Models, methods, concepts & applications of the analytic hierarchy process, vol. 175. 2nd ed. New York: Springer (2012. (International Series in Operations Research & Management Science).

[58] Alonso JA Lamata MT. Consistency in the analytic hierarchy process: a new approach. Int J Uncertain Fuzziness Knowl Based Syst. (2006) 14:445–59. doi: 10.1142/S0218488506004114

[59] Ishizaka A Labib A. Analytic hierarchy process and expert choice: benefits and limitations. OR Insight. (2009) 22:201–20. doi: 10.1057/ori.2009.10

[60] Saaty RW. The analytic hierarchy process – what it is and how it is used. Math Model. (1987) 9:161–76. doi: 10.1016/0270-0255(87)90473-8

[61] Rantakokko M Iwarsson S Mänty M Leinonen R Rantanen T. Perceived barriers in the outdoor environment and development of walking difficulties in older people. Age Ageing. (2012) 41:118–21. doi: 10.1093/ageing/afr136, 22086965

[62] Tsai L-T Rantakokko M Portegijs E Viljanen A Saajanaho M Eronen J . Environmental mobility barriers and walking for errands among older people who live alone vs. with others. BMC Public Health. (2013) 13:1054. doi: 10.1186/1471-2458-13-1054, 24207063 PMC4226209

[63] Maki BE Perry SD McIlroy WE. Efficacy of handrails in preventing stairway falls: a new experimental approach. Saf Sci. (1998) 28:189–206. doi: 10.1016/S0925-7535(98)80008-8

[64] Vidair C Haas R Schlag R. Evaluation of health effects of recycled waste tires in playground and track products. Sacramento (CA): Office of Environmental Health Hazard Assessment, California Environmental Protection Agency (2007).

[65] Wu CC. Impacts of brightness contrast, road environment complexity, travel direction and judgement type on speed perception errors among older adult pedestrians’ road-crossing decision-making. Australas J Ageing. (2024) 43:725–32. doi: 10.1111/ajag.13354, 39037914

[66] Takahashi PY Quigg SM Croghan IT Schroeder DR Ebbert JO. Effect of pedometer use and goal setting on walking and functional status in overweight adults with multimorbidity: a crossover clinical trial. Clin Interv Aging. (2016) 11:1099–106. doi: 10.2147/CIA.S107626, 27621602 PMC5012619

[67] Paragas ED Jr Ng ATY Reyes DVL Reyes GAB. Effects of chromotherapy on the cognitive ability of older adults: a quasi-experimental study. Explore NY. (2019) 15:191–7. doi: 10.1016/j.explore.2019.01.004, 30718190

[68] Ahasan R Imbeau D Astore J Vongchai P Salmoni A Konishi T . Ergonomics of living environment for the people with special needs. J Physiol Anthropol Appl Hum Sci. (2001) 20:175–85. doi: 10.2114/jpa.20.175, 11499165

[69] Choi YO Hwang JH Park JH Shin WS Bang DH. Effects of ramp slope on physiological characteristic and performance time of healthy adults propelling and pushing wheelchairs. J Phys Ther Sci. (2015) 27:7–9. doi: 10.1589/jpts.27.7, 25642025 PMC4305601

[70] Wiener JM Pazzaglia F. Ageing- and dementia-friendly design: theory and evidence from cognitive psychology, neuropsychology and environmental psychology can contribute to design guidelines that minimise spatial disorientation. Healthcare. (2021) 9:929. doi: 10.3390/healthcare9080929, 34047895 PMC8545728

[71] Komisar V Novak AC. Effect of handrail height and age on trunk and shoulder kinematics following perturbation-evoked grasping reactions during gait. Hum Factors. (2023) 65:200–11. doi: 10.1177/00187208211013631, 33945338 PMC9969491

[72] Ulrich RS. Aesthetic and affective response to natural environment In: Altman I Wohlwill JF, editors. Human behavior and environment, vol. 6: Behavior and the natural environment. New York, NY: Plenum Press (1983). 85–125.

[73] Wang Z Liu X Wang X Chen X Xu J Yang Y . Horticultural therapy for general health in the older adults: a systematic review and meta-analysis. PLoS One. (2022) 17:e0263598. doi: 10.1371/journal.pone.0263598, 35143551 PMC8830630

[74] Park SA Shoemaker CA Haub MD. Physical and psychological health conditions of elderly women engaged in gardening activity. HortTechnology. (2016) 26:474–83. doi: 10.21273/HORTTECH.26.4.474

[75] Kelly ME Duff H Kelly S McHugh Power JE Brennan S Lawlor BA . The impact of social activities, social networks, and social support on the cognitive functioning of healthy older adults: a systematic review. Syst Rev. (2017) 6:259. doi: 10.1186/s13643-017-0632-2, 29258596 PMC5735742

[76] Erbino C Toccolini A Vagge I Ferrario PS. Guidelines for the design of a healing garden for the rehabilitation of psychiatric patients. J Agric Eng. (2015) 46:43–51. doi: 10.4081/jae.2015.426

[77] Li N Wang H Fan J. How soundscape perception enhances the well-being of elderly recreational visitors in urban parks: a case study of Zhengzhou people’s Park. Sci Rep. (2025) 15:16652. doi: 10.1038/s41598-025-98125-6, 40360601 PMC12075578

[78] Sowndhararajan K Kim S. Influence of fragrances on human psychophysiological activity: with special reference to human electroencephalographic response. Sci Pharm. (2016) 84:724–51. doi: 10.3390/scipharm84040724, 27916830 PMC5198031

[79] Murroni V Pazzaglia F Meneghetti C De Beni R Gamboz N Borella E. Effectiveness of therapeutic gardens for people with dementia: a systematic review. Int J Environ Res Public Health. (2021) 18:9680. doi: 10.3390/ijerph18189595, 34574519 PMC8469939

[80] Herzog TR Maguire P Nebel MB. Assessing the restorative components of environments. J Environ Psychol. (2003) 23:159–70. doi: 10.1016/S0272-4944(02)00113-5

[81] Zhang X Zhang Y Zhai J Wu Y Mao A. Waterscapes for promoting mental health in the general population. Int J Environ Res Public Health. (2021) 18:11792. doi: 10.3390/ijerph182211792, 34831547 PMC8618438

[82] Lin W Chen Q Jiang M Zhang X Liu Z Tao J . The effect of green space behaviour and per capita area in small urban green spaces on psychophysiological responses. Landsc Urban Plan. (2019) 192:103637. doi: 10.1016/j.landurbplan.2019.103637

[83] Lim Y-S Zoh HD Kim TH Kwon TK. Analyzing the cooling effects of water facilities in urban park: the case of Sangju Namsan Park, South Korea. Atmos. (2024) 15:1456. doi: 10.3390/atmos15121456

[84] Zhu R Yuan L Pan Y Wang Y Xiu D Liu W. Effects of natural sound exposure on health recovery: a systematic review and meta-analysis. Sci Total Environ. (2024) 921:171052. doi: 10.1016/j.scitotenv.2024.171052 38373459

[85] Mu J Wu Y Wang T. Impact of the soundscape on the physical health and the perception of senior adults in senior care facilities. HERD. (2023) 16:155–73. doi: 10.1177/19375867221136234, 36411958

[86] Herzog TR Strevey SJ. Contact with nature, sense of humor, and psychological well-being. Environ Behav. (2008) 40:747–76. doi: 10.1177/0013916507308524

[87] Tong K Thompson CW Carin-Levy G Liddle J Morton S Mead GE. Nature-based interventions for older adults: a systematic review of intervention types and methods, health effects and pathways. Age Ageing. (2025) 54:afaf084. doi: 10.1093/ageing/afaf084, 40207381 PMC11982671

[88] Karakaş N Juri JA. Combating geriatric depression: pet therapy’s revolutionary role and contributions to public health. Arch Depress Anxiety. (2024) 10:52–55. doi: 10.17352/2455-5460.000094

[89] Ulrich RS. View through a window may influence recovery from surgery. Science. (1984) 224:420–1. doi: 10.1126/science.6143402, 6143402

[90] Fancourt D Finn S. What is the evidence on the role of the arts in improving health and well-being? A scoping review. Copenhagen: WHO Regional Office for Europe (2019) Health Evidence Network (HEN) synthesis report 67. 32091683

[91] Huang D Song W Feng R. The impact of age identity on social participation of older adults. BMC Geriatr. (2025) 25:215. doi: 10.1186/s12877-025-05868-y, 40169953 PMC11963424

[92] Völker S Kistemann T. The impact of blue space on human health and well-being—salutogenetic health effects of inland surface waters: a review. Int J Hyg Environ Health. (2011) 214:449–60. doi: 10.1016/j.ijheh.2011.05.001, 21665536

