# Supplementary material for: An Analytic Hierarchy Process-based case study on older adult-friendly community therapeutic landscape design
Source: Front Public Health. 2026 Jan 13;13:1736308. doi: 10.3389/fpubh.2025.1736308 (PMC12834774; doi:10.3389/fpubh.2025.1736308)
Supplement: Supplementary file 1 [file Table_1.DOCX]

**Supplementary Table 1.** Profile of Experts Participating in the AHP Evaluation

| **Expert ID** | **Degree / Position** | **Field of expertise** | **Institution and research background** | **Years of experience** |
| --- | --- | --- | --- | --- |
| Expert 1 | PhD / Professor, Department Chair | Community governance and community studies | Beijing Institute of Fashion Technology; aging-friendly community design; community space | 27 years |
| Expert 2 | PhD / Associate Professor |  | Beijing University of Technology; community space design | 18 years |
| Expert 3 | PhD / Associate Professor, Department Chair |  | Lanzhou University of Technology; urban and community space design research | 25 years |
| Expert 4 | PhD / Lecturer | Public health and health promotion | Beijing Institute of Fashion Technology; therapeutic landscape and sustainable design research | 6 years |
| Expert 5 | PhD / Lecturer |  | Anhui University of Arts; health and sustainable design research | 6 years |
| Expert 6 | PhD / Senior Lecturer | Landscape and urban studies | Shenzhen University; architectural and urban planning design | 11 years |
| Expert 7 | PhD / Researcher |  | Universiti Sains Malaysia; landscape space design | 6 years |
| Expert 8 | PhD / Lecturer |  | Wenzhou University; landscape space planning and design | 8 years |
| Expert 9 | PhD / Researcher |  | Universiti Putra Malaysia; architecture and landscape design | 5 years |
| Expert 10 | Master’s / Researcher | Geriatric medicine and rehabilitation | Beijing University of Chinese Medicine; public health and geriatric medicine | 6 years |
| Expert 11 | PhD / Researcher |  | Kookmin University (Korea); public health and older adults’ health | 5 years |
